# Tackling healthcare providers bias: a systematic review of interventions with implications for inclusive clinical research

**DOI:** 10.1016/j.eclinm.2025.103513

**Published:** 2025-09-19

**Authors:** Maria Grammoustianou, Stefano Maccarone, Emma Gillanders, Gwenn Menvielle, Marie Preau, Stepheline Ginguene, Sibille Everhard, Cyrille Delpierre, Francis Guillemin, Ines Vaz-Luis, Maria Alice Franzoi

**Affiliations:** a Breast Cancer Survivorship Group, INSERM Unit 981, Gustave Roussy, Villejuif, France; b Department for the Organization of Patient Pathways (DIOPP), Gustave Roussy, Villejuif, France; c University of Pavia, Pavia, Italy; d Inserm U1296, Radiations: Santé, Défense, Environnement (Radiation: Health, Defense, Environment), University Lumière Lyon 2, France; e UNICANCER, Direction des Data et des Partenariats, Le Kremlin-Bicêtre, France; f CERPOP, Université Toulouse III-Paul Sabatier, Toulouse, France; g CHRU-Nancy, Inserm, Université de Lorraine, CIC, Epidémiologie Clinique, 54000, Nancy, France; h UMR INSPIIRE, Université de Lorraine, Inserm, Nancy, France

**Keywords:** Implicit bias, Explicit bias, Cultural competence, Disparities, Healthcare providers, Interventions, Digital, Clinical trials, Healthcare

## Abstract

**Background:**

Diverse population inclusion in clinical research is essential for generating high-quality, generalizable data that promotes health equity. However, clinical research often underrepresents elderly individuals, minoritised ethnicities, and marginalized socio-demographic groups. Implicit and explicit biases among healthcare providers can create barriers to equitable participation in clinical trials, affecting patient care, clinical outcomes, and provider-patient trust. Bias-focused interventions have been reported, yet methodological inconsistencies and limited generalizability of studies hinder their effectiveness. This systematic review synthesizes global evidence on interventions designed to address healthcare provider biases, identifying intervention components, trends, outcomes assessed and gaps in the current literature.

**Methods:**

Following PRISMA guidelines, a systematic review of English-language peer-reviewed studies published between 2004 and June 2025 was conducted. The review included both quantitative and qualitative studies assessing interventions targeting healthcare provider biases or cultural competency. A comprehensive search of Scopus, Cochrane, and PubMed MEDLINE databases identified 110 studies. Study quality, risk of bias, and outcomes were assessed using the RoB-2 tool for randomized trials and ROBINS-I for non-randomized studies (PROSPERO ID: CRD42024515985).

**Findings:**

Among the 103 studies assessed for efficacy, 75.7% reported positive outcomes. The majority of studies (69%) were conducted in the U.S. Most interventions combined educational and experiential methods, with the majority being brief (up to 3 h). An increase in the use of digital delivery methods was observed in the past 5 years, with 45% of studies incorporating digital components, particularly after 2020. Key outcomes included explicit bias (53.6%), cultural competency (31.2%), and implicit bias (31.8%), measured mainly by heterogeneous and non-standardized instruments. Notably, only 38 studies (34.5%) assessed actual healthcare provider behavior, and 30 studies (27.2%) evaluated long-term efficacy. High risk of bias and methodological challenges impacted the quality of evidence.

**Interpretation:**

This review highlights positive short-term effects of interventions on implicit bias and cultural competence but suggests future research could benefit from more rigorous trial designs, standardized metrics for measuring efficacy, and actual provider behavior. It also emphasizes the need for more implementation research to identify barriers and facilitators for scaling interventions to ensure their sustainability and broader impact on diversity, equity, and inclusion in healthcare and research.

**Funding:**

This project is funded by WeShare,DIVERSIFY and SIRIC Epicure projects. WeShare program is supported by the French State, managed by the 10.13039/501100001665Agence Nationale de la Recherche under the Investissements Program integrated into France 2030, with reference number: ANR-21-ESRE-0017. DIVERSIFY is funded by La 10.13039/501100004099Ligue Contre le Cancer. SIRIC Epicure is funded by INCA-DGOS-Inserm-ITMO Cancer 18002.

**Research in context**

**Evidence before this study**

Clinical trials often fail to adequately represent diverse populations, limiting the generalizability of findings and exacerbating existing health disparities. Implicit and explicit biases among healthcare providers (HCPs) play a crucial role in this underrepresentation, influencing provider-patient interactions at various stages of care—from prevention and diagnosis to treatment and clinical trial enrollment. These biases can shape the treatment options offered to patients, including eligibility for clinical studies, thereby affecting both patient outcomes and the inclusivity of clinical research.

While previous systematic reviews have explored interventions aimed at reducing biases, most have been limited by outdated information, typically covering studies up to 2021. Given the rapid evolution in this field, our review updates and broadens this scope by capturing the most recent advancements in addressing implicit and explicit biases, alongside enhancing cultural competence. Notably, we explore the increasing use of digital tools and innovative training methods to tackle these issues.

To ensure a comprehensive and current analysis, we conducted a thorough search of Scopus, Cochrane, and PubMed MEDLINE databases for English-language studies published from 2004 to 2024. Key terms such as “healthcare provider bias,” “cultural competence,” and “training interventions” were used to identify relevant articles. Our review focuses on a broad range of medical fields and includes interventions specifically targeting HCP bias and/or cultural competence, with an emphasis on studies that incorporated pre-intervention baseline assessments.

**Added value of this study**

This study provides a comprehensive synthesis of existing interventions, including their components, delivery methods, duration, target populations, and outcomes assessed. It also identifies the strengths and limitations of the current evidence, highlighting gaps in the research. By offering valuable insights into these aspects, the review provides guidance on how to move the field forward, with recommendations at the study design level including population recruitment and outcomes assessed, but also implementation.

**Implications of all the available evidence**

The available evidence suggests a growing focus on addressing healthcare provider biases and cultural competence, with an increase in related research publications. Interventions in this area have demonstrated positive short-term effects, particularly in raising awareness and enhancing cultural understanding. However, gaps remain in assessing the long-term impact of these interventions, especially regarding actual changes in provider behavior. This review highlights the need for more rigorous study designs, including randomized controlled trials and cluster randomizations, to assess efficacy and improve generalizability. There is also a need for standardized outcome measures and greater emphasis on real-world applications, moving beyond provider-reported outcomes to include audits of provider practices. Additionally, more implementation research is critical to identifying the barriers and facilitators to scaling these interventions across diverse healthcare settings. Addressing these challenges in future research could present an opportunity to drive more sustainable and evidence-based progress in diversity, equity, and inclusion in healthcare and research.

## Introduction

The inclusion of diverse populations in clinical research is essential for generating high-quality data that is generalizable and applicable to real-world situations. Moreover, it fosters health equity by ensuring equal opportunities for all individuals to participate in clinical trials.^1^, ^2^, ^3^ Despite this, research populations in oncology and other healthcare fields frequently fail to reflect the diversity of the general population.^4^ Clinical trials often underrepresent women, elderly patients, individuals with comorbidities, and marginalized racial and sociodemographic groups, despite increasing concerns among policymakers, patient advocates, medical societies, and some industry leaders.^1^^,^^5^^,^^6^ In the United States, only 4%–6% of clinical trial participants are Black, and 3%–6% are Hispanic.^4^ Additionally, 20–30% of participants are aged 65 years or older, and 20% reside in rural areas.^4^ A recent European systematic review of 97 phase II and III clinical trials for breast cancer (involving 113,045 patients) found that 92.65% of participants were White, 1.08% were Asian, 0.88% were Black, and 3.20% identified as Hispanic/Latino.^7^ In France, several studies have identified various barriers to participation in cancer research. Specifically, socioeconomically disadvantaged groups,^8^^,^^9^ those with limited health literacy,^10^ older adults,^11^ and individuals with lower financial resources, rural residents, and patients treated at non-comprehensive centers are less likely to be invited to participate in clinical trials.^12^ These factors contribute to the underrepresentation of certain populations in research and highlight the need for more inclusive and accessible trial designs.^10^^,^^12^^,^^13^

Patient-physician communication plays a crucial role in clinical trial decision-making, with patients often relying on physicians as their most trusted source of information.^14^ However, data from both Europe and the United States reveal that only about 10% of patients are invited to participate in clinical trials, with invitation rates being particularly low for disadvantaged groups.^15^^,^^16^ For example, the VICAN study, conducted in France found that younger patients, those with higher health literacy, and those treated in comprehensive cancer centers were more likely to receive invitations. Importantly, once invited, 75%–80% of patients agree to participate, regardless of their health literacy or race.^10^ Similar findings were reported in a 2020 American meta-analysis, which showed that more than half of patients accepted participation when offered, with slightly higher participation rates observed among non-white patients.^7^

One potential reason for unequal access to clinical studies may be linked to both implicit and explicit bias among healthcare providers. These biases have been shown to result in worse clinical outcomes, negatively affect patient care and quality of life, and undermine patient-provider trust.^17^, ^18^, ^19^, ^20^, ^21^, ^22^, ^23^ Explicit bias refers to conscious attitudes and beliefs toward different patient groups, while implicit bias involves unconscious associations that influence healthcare provider's judgments and behaviors, often without their awareness. These biases are frequently shaped by societal norms and cultural stereotypes.^12^ Cultural competence has emerged as a critical approach to addressing these biases. Initially focused on ethno-cultural characteristics, cultural competence now takes a broader perspective, understanding culture as a dynamic, multilayered concept shaped by both political and social contexts.^24^^,^^25^

Numerous studies, which our study plans to systematically review, have been conducted to evaluate interventions aimed at improving cultural competency and reducing healthcare provider bias. However, many of these interventions are institution-specific or concentrated in particular countries or healthcare settings, which introduces significant variability in the evidence. This makes it difficult to generalize findings across diverse healthcare environments and settings. Furthermore, the quality of evidence supporting these interventions is often unclear, as many studies suffer from methodological limitations, such as inconsistent designs and lack of standardization in outcomes. To address these challenges, we sought to conduct a systematic review to synthesize the existing evidence, identify common patterns in intervention components and delivery methods, and assess the effectiveness of different strategies across varied healthcare contexts, including outside the research setting. Additionally, we aimed to pinpoint gaps in the current literature, highlight areas requiring further research, and guide the development of more standardized, scalable solutions that can enhance equity, inclusion, and diversity in healthcare and clinical research.

This systematic review aims to assess available interventions aiming to reduce implicit bias in research and healthcare and their potential efficacy in changing knowledge and attitudes towards more objective clinical decision-making, increasing equity in healthcare delivery and in research participation. Specifically, the results of this review will be used to inform the development of bias-focused interventions within a toolkit designed to improve engagement, diversity, equity, and inclusion in clinical research, with a particular focus on oncology.^26^

## Methods

The protocol for this systematic review was registered and approved for publication in the International Prospective Register of Systematic Reviews (PROSPERO ID: CRD42024515985).

### Search strategy

Search techniques were employed in Scopus, Cochrane, and PubMed MEDLINE databases to capture English-language publications from 2004 to 2024, ensuring the inclusion of the most current research outcomes and evidence. Medical Subject Headings (MeSH) and key terms from related articles were analyzed to identify relevant search terms (see Supplementary Material: Search string strategy). Particular emphasis was placed on the fields of oncology and clinical research, given that this project constitutes a preliminary investigation intended to inform the development of a training program aimed at reducing implicit bias in oncological clinical trial. We also tracked references from included studies and relevant reviews to uncover additional studies. Searches were re-run just before the final analysis to incorporate any newly identified studies, the last search was performed on December 31st 2024 and an additional search has been performed on June 15th 2025.

### Selection criteria

This systematic review includes peer-reviewed studies that assess interventions aimed at reducing healthcare provider biases or improving their knowledge, skills, or attitudes related to cultural competency. Both quantitative studies (such as cohort and randomized trials) and qualitative studies were considered. Only English-language studies involving adult populations (18+) that included a pre-intervention baseline assessment (measured either qualitatively or quantitatively) were included. Excluded from this review were opinion pieces, editorials, letters to the editor, dissertations, book chapters, study protocols, and case reports.

Studies from all medical fields and care settings were considered, with comparators being either control groups or participants' pre-intervention status. Outcomes were measured both pre- and post-intervention, either qualitatively or quantitatively. Studies focusing on students (in the healthcare field, such as medical, nursing, etc.) were excluded, as evidence suggests that students typically exhibit lower levels of bias and higher receptivity to interventions compared to more experienced healthcare professionals, who may resist change due to ingrained biases developed over prolonged exposure in the workplace.^21^^,^^22^

### Data extraction

The systematic review was conducted following the guidelines outlined in the Preferred Reporting Items for Systematic Review and Meta-Analysis Protocols (PRISMA-P) Statement.^27^ Two reviewers (MG, EG) independently screened records for study selection and data extraction. Both reviewers assessed records for inclusion using titles and abstracts, and if the inclusion or exclusion of a study was not clear from the abstract, the full article was reviewed. Any conflicts during the primary study selection process were resolved collaboratively between the two reviewers. In cases where conflicts remained unresolved, a third experienced researcher (MAF) was consulted to make a final decision.

A data extraction form was developed in Excel to collect key variables, including: study characteristics (author, title, year, country, number of participants, design, setting, outcomes), population demographics (age, sex, race, ethnicity, profession), intervention details (type, components, duration, frequency, delivery method, comparator groups, outcomes), and reported outcomes (changes in awareness, bias, behavior, cultural competence, and feasibility metrics such as utility and satisfaction).

### Assessment of study quality

The risk of bias for each study included in the analysis was assessed individually by two independent reviewers using the RoB-2 tool (Revised Cochrane Risk of Bias tool for randomized trials) for randomized clinical trials, and the ROBINS-I tool (Risk Of Bias In Non-Randomized Studies) for non-randomized studies. Any disagreements between the reviewers were resolved through discussion, and, if necessary, a third review author was involved to resolve outstanding conflicts.

### Definitions of outcome categories

For data analysis purposes, we defined the following items among the outcomes of interventions:

a) **“Implicit bias”**: This refers to unconscious processes such as stereotypes and prejudices that influence decision-making and behavior.
b) **“Explicit bias”**: This involves overt expressions of bias, including stigma, social distance, behaviors, and attitudes that are consciously expressed.
c) **“Cultural competency”**: This focuses on the ability to provide culturally sensitive care, incorporating constructs such as cultural awareness, knowledge, and sensitivity.
d) **“Skills”**: Outcomes related to practical abilities, including communication and clinical skills that are relevant to patient care. (For definitions and further details, see Supplementary Material: Glossary of relevant terms).

### Data analysis

A descriptive analysis of the included studies was conducted to examine the healthcare context, country of origin, intervention type, duration, delivery method, target population characteristics, outcomes, and assessment tools used.

Given the wide range of outcomes measured across the studies, we anticipated limited scope for meta-analysis. According to the study protocol (PROSPERO ID: CRD42024515985), a pooled analysis was planned for studies that used the same type of intervention, comparator, and outcome measure. However, due to high levels of heterogeneity, differences in populations, and varying outcomes assessed, we determined that a pooled analysis would be inconclusive or too biased to report.

### Role of the funding source

The funders had no role in the design, execution, analysis, or interpretation of the data presented in this review.

## Results

**Fig. 1:**
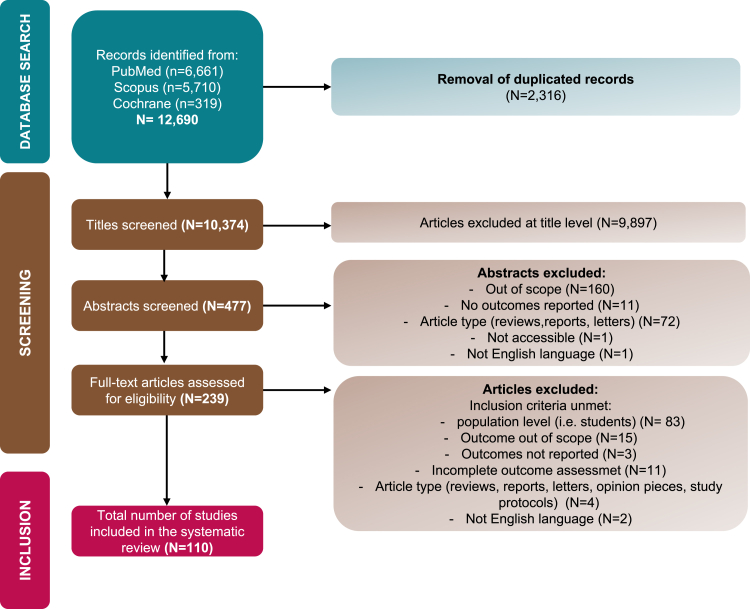
Search and study selection for the systematic review according to PRISMA guidelines.

Among 12,690 studies screened, 104 studies were finally included in the systematic review. Study selection process is detailed in the PRISMA flow diagram exposed in Fig. 1. Information on all studies included in this systematic review is provided in the Supplementary Material—Table S1: Summary of Included Studies.

### Study and participant characteristics

A summary of the study and intervention characteristics included in this review is provided in Table 1. Most studies were conducted in the United States (69%, n = 76), followed by Europe (11%) and East Asia (10%), with the majority using non-randomized quasi-experimental designs (73.6%, n = 81). The remaining studies were randomized controlled trials (19.1%) and feasibility studies (5.4%).

**Table 1:** Summary of studies and interventions’ characteristics.

Intervention characteristics	Studies (n)	Category	n (%)
Study location	110	USA	76 (69)
		EU	12 (11)
		Eastern Europe	1 (1)
		East Asia	11 (10)
		Africa	2 (1.8)
		Australia	5 (4.5)
		Latin America	1 (1)
		West Asia	2 (1.8)
Year of publication	110	After 2021	28 (25.45)
		Between 2017 and 2021	44 (40)
		Between 2012 and 2016	28 (25.45)
		Before 2011	11 (10)
Study type	110	Non Randomized, Quasi-Experimental	81 (73.6)
		Randomized Controlled Trials	21 (19.1)
		Feasibility Studies	6 (5.4)
Study focus	110	Cultural Competence	23 (20.9)
		Healthcare Disparities	19 (17.2)
		Race/Ethnicity Bias	22 (20)
		Sexuality/Gender Bias	15 (13.6)
		Mental Health Bias	14 (12.7)
		HIV-related Bias	7 (6.3)
		Weight Bias	6 (5.4)
		Ageism	2 (1.8)
		Substance abuse related Bias	2 (1.8)
Study context	110	General Healthcare	84 (76.3)
		Oncology	9 (8.2)
		Emergency Medicine	10 (9)
		Perinatal Health	4 (3.6)
		Mental Health	1 (0.9)
		Paediatrics	1 (0.9)
		Sexual and Reproductive Health care	1 (0.9)
Intervention type	110	Educational and Experiential	55 (50)
		Purely Educational	35 (32)
		Purely Experiential	20 (18)
Delivery method	110	In-Person	73 (60)
		Digital	31 (24)
		Both Methods	6 (16)
Intervention duration	96	Up to 3 h	42 (43.7)
		1 day to 1 week	28 (29.1)
		>1 week	23 (24.0)
		>1 month	3 (3.1)
Intervention pace	109	Synchronous	102 (93.6)
		Self-Paced	5 (4.6)
		Both	2 (1.8)
Intervention frequency	103	Single Session	71 (68.9)
		Multiple Sessions	29 (28.1)
		Both options	3 (2.9)
Boost session	110	Yes	2 (1.8)
		No	108 (98.2)
Outcomes evaluated	110	Explicit Bias	59 (53.6)
		Cultural Competency	35 (31.2)
		Skills	34 (30.9)
		Implicit Bias	35 (31.8)
		Patient Outcomes	7 (6.3)
		Multiple Outcomes Assessed	69 (62.7)

Characteristic: The study attribute being analysed (e.g., Study Location, Study Type).

Studies (n): Total number of studies that evaluated this characteristic.

Category: Specific subgroups within each characteristic (e.g., USA, Europe, Non-Randomized).

n (%): Number and percentage of studies in each category.

**Fig. 2:**
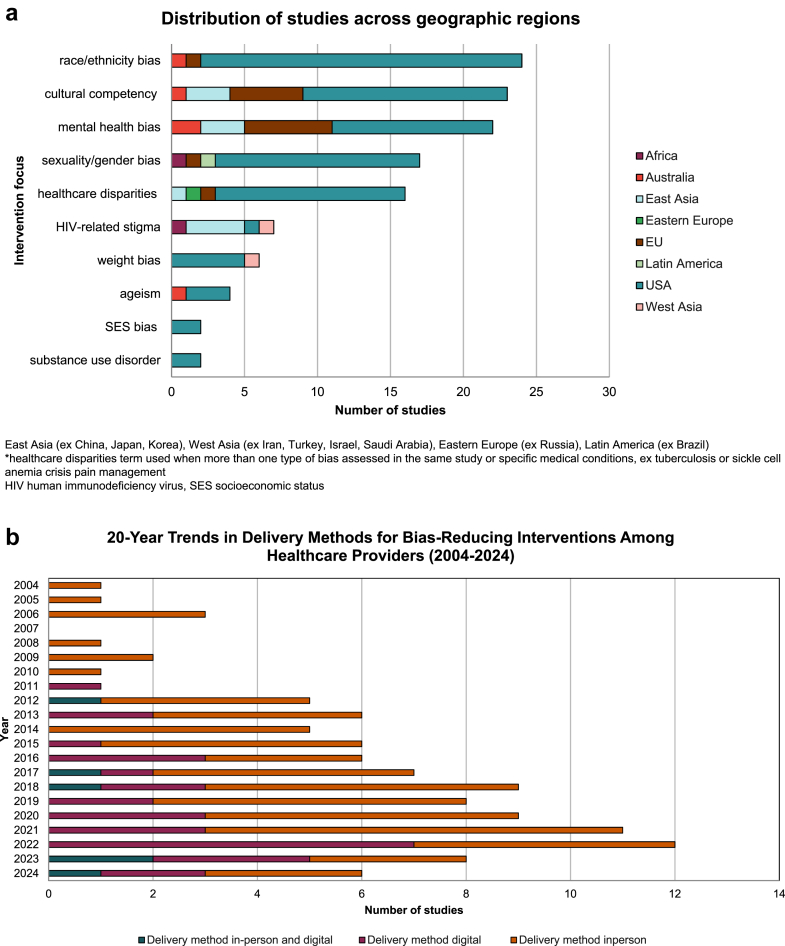
a) Distribution of studies per geographic location and intervention focus. b) Trends in intervention delivery method for bias reduction interventions over the last 20 years.

Key intervention focus areas included cultural competence (20.9%, n = 23), healthcare disparities in general, where multiple types of bias were evaluated in the same study (17.2%, n = 19), or one specific type of bias such as race/ethnicity bias (20%, n = 22) (Fig. 2). Different types of bias were prioritized across countries, reflecting variations in cultural contexts and healthcare system factors. For example, in African and Asian countries, there is a focus on HIV-related stigma, a concern less prevalent in Western countries, where racial and cultural competency biases dominate (Fig. 2a).

Most studies (76.3%) were not focused on a single specialist sub-population, involving healthcare providers in general. The most common fields of study following these interventions were Emergency Medicine (9%), Oncology (8.2%), Perinatal Health (3.6%), and lastly, Mental Health, Sexual and Reproductive Healthcare, and Pediatrics, all with only one study (Table 1).

Only four studies evaluated interventions aimed at reducing bias in clinical research, all of which were conducted in the United States and within the oncology setting (See Supplementary Material Table S2: Interventions focusing on clinical trials). More specifically, two of these studies focused on cultural competence,^28^^,^^29^ while the remaining two addressed racial, ethnicity, and socioeconomic status bias.^30^^,^^31^ Three of the studies interventions were delivered digitally.^29^, ^30^, ^31^

Table 2 describes the main characteristics of healthcare providers included in the studies. With regards to gender distribution, (n = 84, HCPs = 11,629), 75.9% of participants were female (n = 8823) and 24.1% male (n = 2806). Among studies providing racial and ethnicity data (n = 51, HCPs = 9320), 54, 4% of healthcare providers were identified as White (n = 5076), followed by Asian (20.03%, n = 1867), Black/African (11.66%, n = 1087), Hispanic/Latinx (8.38%, n = 781), and other racial groups (5.46%, n = 509). The majority of studies (75%) included mixed professional specialties, which consisted of both clinicians (physicians and non-physicians) and non-clinicians (n = 11,563). Twenty-four studies focused only on physicians (n = 1017), 19 included only non-physician clinicians (n = 1387), and 4 studies exclusively targeted non-clinicians (n = 1431). Among the 39 studies reporting healthcare providers’ years of work experience, 45% had over 10 years of experience, 37% had between 5 and 10 years, and 18% had less than 5 years.

**Table 2:** Characteristics of Healthcare Providers participating in the interventional studies.

Population characteristics	Studies (n)	Category	% in studies	Participants (n)
Gender	84	Female	75.9	8823
		Male	24.1	2806
Race/Ethnicity	51	White	54.46	5076
		Black/African	11.66	1087
		Asian	20.03	1867
		Hispanic/Latinx	8.38	781
		Other	5.46	509
Population type	110	Experienced HCPs	92.1	13,915
		In-training HCPs	2.5	385
		Mixed Populations	5.3	803
Specialty of participants	110	Clinicians and Non-Clinicians	75	11,563
		Clinicians (Physicians/Non-Physicians)	6.6/9.01	1017/1387
		Non-Clinicians	9.31	1434
Years of work experience	38	>10 years	36.6	1288
		5–10 years	49.9	1756
		<5 years	23.5	476

Population Characteristic: The demographic factor analyzed (e.g., Gender, Race/Ethnicity).

Studies Evaluated: Number of studies reporting on this characteristic.

Category: Specific subgroups within each characteristic (e.g., Female, White, Asian).

% in Studies: Percentage of each category within studies that evaluated this characteristic.

Participants (Total %): Number and percentage of participants in each category within studies evaluating the characteristic.

### Intervention characteristics

**Fig. 3:**
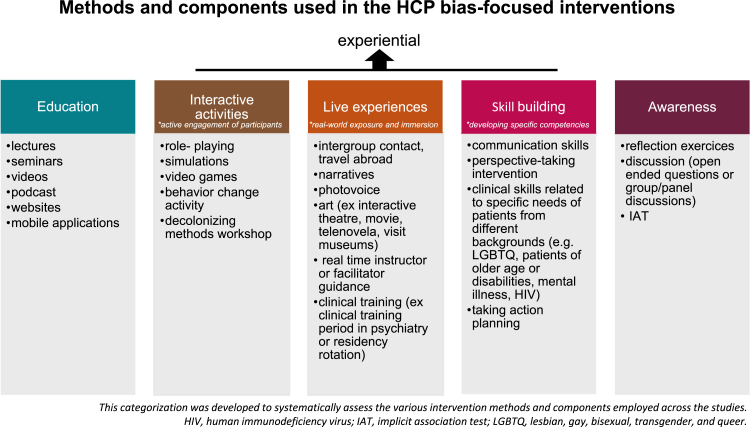
Methods and components used in healthcare provider-focused interventions.

Following a thorough analysis, we identified three main categories of intervention methods: educational, experiential, and awareness-enhancing methods (Fig. 3). Approaches designed to increase awareness, such as reflection exercises and post-intervention discussions, were incorporated into nearly all training programs but were never used as standalone methods. As a result, these techniques were not evaluated separately in the studies. Educational training programs generally involved passive forms of training, while experiential methods referred to training processes requiring active participation. Experiential methods were further subdivided into three subgroups:

i) **Interactive activities** involving methods that lead to the active engagement of participants, such as role-playing or decolonizing method workshops;
ii) **Live experiences** providing real-world exposure and immersion, allowing participants to experience different contexts directly, such as intergroup contact or narrative-based activities;
iii) **Skill-building** focusing on developing specific competencies directly applicable to clinical practice, such as communication skills or perspective-taking interventions.

This categorization was developed to systematically synthesize the various approaches used in bias-focused interventions (Fig. 3). Interventions most commonly (51%) combined both educational and experiential methods^24^, ^25^, ^26^, ^27^, ^28^, ^29^, ^30^, ^31^, ^32^, ^33^, ^34^, ^35^, ^36^, ^37^, ^38^, ^39^, ^40^, ^41^, ^42^, ^43^, ^44^, ^45^, ^46^, ^47^, ^48^, ^49^, ^50^, ^51^, ^52^, ^53^, ^54^, ^55^, ^56^, ^57^, ^58^, ^59^, ^60^, ^61^, ^62^, ^63^, ^64^, ^65^, ^66^, ^67^, ^68^, ^69^, ^70^, ^71^, ^72^, ^73^, ^74^, ^75^, ^76^, ^77^, ^78^, ^79^, ^80^, ^81^, ^82^, ^83^, ^84^, ^85^, ^86^, ^87^, ^88^, ^89^, ^90^, ^91^, ^92^, ^93^, ^94^, ^95^, ^96^, ^97^, ^98^ while a lesser proportion consisted of purely educational^28^^,^^29^^,^^34^, ^35^, ^36^, ^37^, ^38^, ^39^, ^40^, ^41^, ^42^, ^43^, ^44^, ^45^, ^46^, ^47^, ^48^, ^49^, ^50^, ^51^, ^52^, ^53^, ^54^, ^55^, ^56^, ^57^, ^58^, ^59^, ^60^, ^61^, ^62^, ^63^ or purely experiential^64^, ^65^, ^66^, ^67^, ^68^, ^69^, ^70^, ^71^, ^72^, ^73^, ^74^, ^75^, ^76^, ^77^, ^78^, ^79^, ^80^ training tools (29.8% and 19.2% respectively). With respect to intervention duration, among studies providing this information (n = 90), the majority of interventions were brief, lasting up to 3 h (44%, n = 40), 30% lasted between 1 day and 1 week, and 23 studies (25.6%) analyzed interventions that lasted longer than a week. Furthermore, the vast majority of studies reporting intervention frequency (n = 97) were performed as single sessions (72%), while 25% of interventions were structured through multiple sessions, and 3 studies (3%) allowed for both options.

Regarding intervention delivery methods, the use of digital interventions increased significantly over the past decade (Fig. 2b), with more than half (53%) of all studies featuring a digital component being published after 2020.

### Outcomes and assessment methods

Multiple and heterogeneous outcomes were assessed across the studies. In order to provide a synthesis of the main outcomes evaluated in bias-reduction interventions, we organized them into five distinct categories (See Supplementary Material Figure S1: Outcomes assessed). Each category represents a dimension of bias or skillset relevant to healthcare providers' interactions with patients.

Each study evaluated at least one of the following outcomes: explicit bias (53.6%), cultural competency (31.2%), skills (30.9%), implicit bias (31.8%), and patient outcomes (6.3%),^29^^,^^52^^,^^70^^,^^81^, ^82^, ^83^ with 62.7% of the studies assessing more than one of these outcomes simultaneously. Feasibility was assessed in one-third of the studies. A longitudinal analysis of outcome trends revealed a notable rise in studies addressing explicit bias, with continued attention to cultural competency as a key area of research (See Supplementary Material Figure S2: Trends of outcomes assessed over the years).

Various assessment instruments were employed across the reviewed studies to measure different types of bias and competencies among healthcare providers (See Supplementary Material: Table S1—Summary of Included studies), with the choice of instrument depending on the specific bias evaluated. However, most studies used non-validated questionnaires or open-ended questions, limiting the standardization of assessment methods. Many studies incorporated more than one measurement method to capture a more nuanced assessment of one outcome or to evaluate different outcomes linked to the intervention (e.g., impact of the intervention on explicit and implicit bias).

Approximately one third of studies (n = 38) evaluated interventions for their feasibility, with most interventions considered feasible due to their applicability, acceptability, and participant satisfaction. Among the 103 studies assessed for efficacy, 75.7% were considered to have improved their pre-defined outcomes, while 24.3% did not show improvement. Of the 110 studies reviewed, only 30 assessed the long-term efficacy of interventions with follow-ups conducted at variable intervals rather than consistent time points, (e.g. one, two, three, or six months post-intervention).^24^^,^^26^^,^^27^^,^^31^^,^^32^^,^^35^^,^^38^^,^^46^^,^^50^, ^51^, ^52^^,^^54^^,^^59^^,^^64^^,^^65^^,^^70^^,^^71^^,^^73^^,^^77^^,^^99^, ^100^, ^101^, ^102^, ^103^

**Fig. 4:**
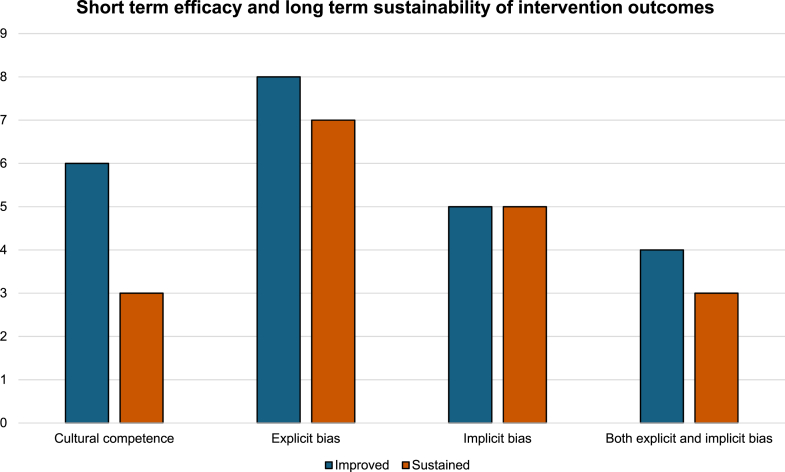
Short term efficacy and long term sustainability of intervention outcomes.

To enhance comparability across heterogeneous studies, intervention efficacy was classified using a binary framework; studies exhibiting only partial improvements across outcomes were not deemed fully effective. Fig. 4 includes only those studies that demonstrated consistent improvements and sustained long-term effects, thereby ensuring alignment with the assumptions of the statistical models. While this binary classification necessarily simplifies a more nuanced reality, it was supplemented with a descriptive analysis (Supplementary Table S1) to preserve interpretive depth.

Notably, patterns emerged that merit mention. Implicit bias appeared harder to shift compared to explicit bias or cultural competency. However, when improvements in implicit bias did occur, they tended to be more durable over time. By contrast, explicit bias and cultural competence outcomes improved more easily following intervention but showed less consistency in long-term maintenance. This may suggest that sustained effects, particularly for more surface-level constructs, depend on repetition or reinforcement over time.

Among the initially effective studies (n = 24), most targeted explicit bias (33%) or cultural competency (25%) and implicit bias (25%), with 87.5% of explicit bias interventions and 50% of cultural competency interventions maintaining effects at follow-up. However, the smaller number of studies focused on implicit bias limits the strength of inference, and this pattern should be interpreted cautiously given key limitations: the overall number of studies remains small, there is substantial heterogeneity in outcome measures and follow-up intervals, and the optimal timing for follow-up or intervention repetition remains unknown. Still, the observed trends warrant attention and may help guide future research and intervention design. When considering the studies targeted at clinical trials, all of them reported improvement in their pre-defined outcomes, with the specific exception of healthcare provider's knowledge and attitudes toward clinical trials in Michaels et al.’s study.^28^ Furthermore intervention assessing feasibility (n = 2) were considered feasible, and it is worth noting Wells et al.’s success in increasing minority patients' accrual rates.^29^

### Quality assessment of studies

The quality of evidence was affected by a high risk of bias, particularly in non-randomized studies, due to confounding factors, participant selection bias, and missing data (See Supplementary Material Table S3: Risk of bias of non-randomized trials using ROBINS I tool). While randomized trials had lower bias, they still faced challenges such as missing data and deviations from protocols (See Supplementary Material Table S4: Risk of bias of randomized trials using RoB2 tool).

## Discussion

This systematic review provides a comprehensive overview of ongoing initiatives aimed at addressing healthcare provider-related bias in healthcare and research. While prior systematic reviews have been conducted in this area, their search criteria were limited to studies published up to 2021.^100^^,^^104^^,^^105^ As this field continues to evolve, many relevant studies have emerged since then (n = 28). Unlike prior reviews, the present work includes not only interventions directly targeting bias but also those focused on cultural competence. Furthermore, it expands the geographic scope by incorporating studies conducted outside North America.

Our findings reveal a notable increase in studies addressing healthcare providers' biases over the past two decades, underscoring the growing emphasis on bias reduction and cultural competence in multiple healthcare and research settings. However, most studies remain concentrated in the United States, with only a few conducted in Europe, Latin America, Africa, and Asia. This geographic concentration underscores the need for greater global awareness of implicit bias, as healthcare disparities and bias-related outcomes have been documented worldwide.^17^^,^^101^^,^^105^, ^106^, ^107^, ^108^, ^109^, ^110^ In response, ongoing initiatives in Europe, such as the Innovative Health Institute's READI consortium, aim to address this gap by promoting diversity, inclusion, and equity in clinical trials.^111^ The READI consortium, which unites 73 organizations across 18 European countries, seeks to enhance the understanding of diversity and underrepresented communities across Europe by promoting population representativeness in clinical research. This initiative will enable researchers and industry stakeholders to better understand the impact of study design on patient recruitment through use cases.

It is important to acknowledge that structural and systemic barriers—limited funding, editorial bias and the undervaluation of locally situated knowledge—may continue to restrict scholars in Latin America, Africa and parts of Asia from publishing on implicit bias.^102^^,^^112^, ^113^, ^114^ In resource constrained contexts, scarce research budgets are understandably channelled toward pressing public health priorities such as maternal mortality reduction, immunisation and HIV control, leaving little capacity for work on implicit bias.^115^^,^^116^ As a result, awareness of implicit bias exists but opportunities to conduct, implement and disseminate related studies are curtailed, producing an uneven evidence base that sidelines Global South perspectives.^117^^,^^118^

Any call for greater global action on implicit bias must therefore confront these asymmetries by embedding equitable authorship, fair funding mechanisms and recognition of diverse epistemologies into research infrastructures.^103^^,^^119^^,^^120^

In the oncology field, the WeShare Consortium in France is leveraging digital health tools to enhance diverse and equitable participation in clinical trials.^26^ These tools include decentralized and remote patient engagement platforms, real-time diversity and inclusion metrics, and inclusive pathways such as working with digital navigators and providing multilingual materials. Additionally, the development of a digital training program aimed at addressing healthcare providers' implicit bias is planned, with this systematic review serving as the first step in identifying existing interventions to adapt to the European context.

This shift towards digital solutions is particularly significant, as the review highlights the growing adoption of digital tools for delivering cultural competence and bias training, offering a broader reach and more personalized learning experiences for healthcare providers. Research suggests that electronic continuing medical education may be more effective than traditional educational methods—such as lectures or static text—in improving healthcare1 providers’ knowledge, adherence to clinical guidelines, and patient outcomes.^121^^,^^122^

**Fig. 5:**
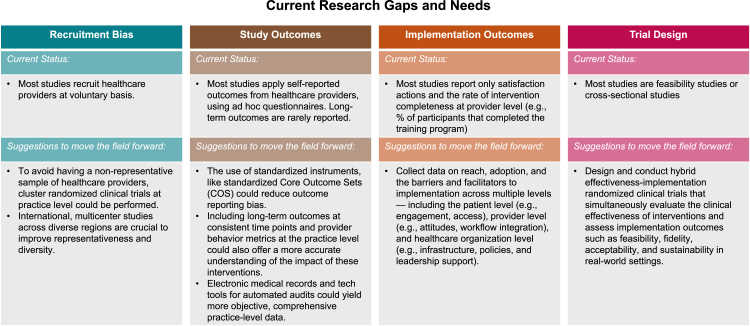
Current research gaps and suggestions to move the field forward.

While this review identifies several promising interventions, nearly all of which demonstrate positive feasibility and efficacy outcomes, much work remains in refining how studies on bias interventions for healthcare providers are planned and evaluated (Fig. 5).

First, most of the studies included did not employ a randomized clinical trial design, meaning they were not optimally powered to assess efficacy outcomes. This limitation underscores the need for more rigorously designed trials to better evaluate the true effectiveness of interventions.

Secondly, while a strength of the review lies in its inclusion of study populations of different healthcare specialists and work experience—comprising both experienced and less experienced healthcare providers, as well as clinicians and non-clinicians involved in the healthcare pathway—the study populations still often lacked diversity. Most participants were predominantly white and female, which limits the generalizability of the results. Additionally, voluntary participation may attract more motivated individuals, which could lead to inflated positive outcomes and may not reflect the broader healthcare workforce.

To address these concerns, cluster-randomized trials^123^ at the level of clinical practices or healthcare centers, rather than at the individual provider level, could help mitigate biases in participant selection. This approach would allow for a more representative sample, potentially improving the external validity of the findings and offering a more accurate assessment of intervention efficacy in real-world settings.

As previously noted, there is a critical need for international, multicenter studies conducted across diverse geographic and cultural settings. Such studies are key to addressing concerns about the lack of representativeness in many clinical trials, improving the generalizability of findings, and promoting more equitable global healthcare outcomes.

Third, the studies assessed a broad range of outcomes, many of which remained heterogeneous even after stratification by category, with most studies evaluating multiple outcomes simultaneously. This variability highlights the need to standardize efficacy metrics for bias-focused interventions to better define and compare their benefits. While self-reported validated tools, such as the Implicit Association Test (IAT), can be used, their reliability is limited given the complex context of bias and stereotypes.^124^ Therefore, including outcomes related to actual provider behavior,^55^^,^^83^^,^^88^ rather than relying solely on self-reported measures, could offer a more accurate understanding of the impact of these interventions on healthcare disparities.

This could be achieved through the retrieval of administrative data, such as actual care and research decisions, medical referrals, and research inclusion logs.^125^, ^126^, ^127^, ^128^ Leveraging electronic medical records and technology tools for automated auditing and data collection at the practice level could provide more objective and comprehensive data, allowing for a clearer assessment of the interventions' real-world effects on healthcare inequalities.

An additional strategy that has been advocated involves the establishment of an international consensus on a standardized core outcome set (COS) to be consistently reported across all clinical trials conducted within this domain. The aim of this approach is to enhance comparability, reduce outcome reporting bias, and improve the synthesis of evidence across studies. In support of this effort, the COSMIN (COnsensus-based Standards for the selection of health Measurement INstruments)^129^ initiative offers a comprehensive methodological framework for the development and evaluation of such core outcome sets, defined as “a consensus-based agreed minimum set of outcomes that should be measured and reported in all clinical trials of a specific disease or trial population; it is a recommendation of what should be measured and reported in all clinical trials”.

Moreover, while indeed, positive changes in healthcare providers' attitudes and knowledge have been documented in the studies included in this review, and evidence suggests that these can positively affect clinical care,^130^ most studies focused on short-term efficacy, typically measuring outcomes immediately after the intervention. This limited timeframe makes it difficult to assess the long-term sustainability of these interventions. Even when long-term outcomes were evaluated, the time points were inconsistent across studies, further complicating the ability to draw clear conclusions. To enhance the robustness of future studies, it is crucial to incorporate consistent and standardized measures of long-term efficacy. This would provide valuable insights into the lasting impact of these interventions and could inform necessary adjustments, such as the inclusion of booster training or follow-up sessions, to sustain the benefits over time.

Finally, although feasibility of the interventions has been demonstrated, a more detailed implementation research^131^^,^^132^ is needed to understand the barriers and facilitators that influence the reach, adoption, efficacy, and sustainability^133^ of these interventions at the provider, patient, and healthcare organization levels.^134^ Investigating how to effectively disseminate these interventions across diverse healthcare settings and practices will be essential for their widespread implementation.

In conclusion, this systematic review highlights the growing body of evidence surrounding implicit bias and cultural competence interventions for healthcare providers. While significant progress has been made, particularly with the increasing use of digital tools and global initiatives, there remain critical gaps in the research. Most studies are concentrated in North America and often suffer from limited diversity in participant populations. To advance the field, future research must focus on more diverse, globally representative samples, standardized efficacy metrics, and long-term outcomes. Indeed, advancing global understanding of implicit bias requires acknowledging and addressing the structural barriers that limit researchers from the Global South. A truly inclusive approach must recognize these inequities and support greater participation from underrepresented regions in shaping this critical field. Additionally, implementation research is needed to understand how to best integrate these interventions into real-world healthcare settings, ensuring they are effectively adopted and sustained across different regions and healthcare systems. To this end, developing an international consensus on a core outcome set is key to improving consistency, reducing bias, and strengthening evidence synthesis in clinical trials. By addressing these challenges, we can better equip healthcare providers to reduce implicit bias, improve patient outcomes, and foster a more inclusive and equitable healthcare environment.

## Contributors

Conceptualization: Maria Alice Franzoi (MAF); Data curation and formal analysis: Maria Grammoustianou (MG); Funding acquisition: MAF; Investigation: MG, EG; Methodology and Software: MG; Project administration and Supervision: MAF; Accessed and Verified Data: Maria Grammoustianou (MG), Stefano Maccarone (SM) Visualization and Writing—original draft: MG, Writing—review & editing: Stefano Maccarone (SM), Gwenn Menvielle (GM), Marie Preau (MP), Stepheline Ginguene (SG), Sibille Everhard (SE), Cyrille Delpierre (CD), Francis Guillemin (FG), Ines Vaz-Luis (IVL), EG, MG, MAF. All authors read and approved the final manuscript.

## Data sharing statement

Data from this systematic review is available from the first author upon reasonable scientific request.

## Declaration of interests

Maria Grammoustianou: None, Maria Alice Franzoi: Research funding from Resilience Care (Institution); Gilead Sciences (Institution); Speaker Honoraria from Novartis, Emma Gillanders: None; Maccarone Stefano: None; Francis Guillemin: None; Preau Marie: None; Ginguene Stepheline: None; Gwenn Menvielle: None; Cyrille Delpierre: None; Ines Vaz-Luis: Research funding from Resilience Care (Institution); Speaker Honoraria from: Agmen, Astra Zeneca, Pfizer/Edimark, Novartis, Sandoz, Chugal Pharmaceutical, Servier Monde, Institut de recherches internationales Servier, Doctaforum Medical Marketing SL.
