# Supplementary material for: Tackling healthcare providers bias: a systematic review of interventions with implications for inclusive clinical research
Source: eClinicalMedicine. 2025 Sep 19;89:103513. doi: 10.1016/j.eclinm.2025.103513 (PMC12495421; doi:10.1016/j.eclinm.2025.103513)
Supplement: Supplementary Material [file mmc1.docx]

**Enhancing Real-World Representation in Clinical Trials: Tackling Healthcare Providers’ Bias – A Systematic Review**

**SUPPLEMENTARY MATERIAL**

**Supplementary file 1. Glossary of relevant terms**

| Implicit (unconscious) bias | A negative attitude, of which one is not consciously aware, against a specific social group, that affect our understanding, actions, and decisions. (1) |
| --- | --- |
| Explicit bias | Attitudes and beliefs individuals consciously acknowledge; can be directly assessed through self-report measures and often underpin overtly discriminatory actions. (1) |
| Cultural competence | A set of congruent behaviors, attitudes and policies that come together to enable work in cross-cultural situations; cognitive, affective, behavioural, linguistic skills for communication with people of other cultures.(2) |
| Cultural awareness | Self-examination of personal biases and the recognition that cultural competence is a continuous learning process.(2) |
| Cultural desire | A genuine commitment and passion for caring for diverse populations, driven by an intrinsic motivation to achieve cultural competence.(2) |
| Cultural encounters | Direct interactions with patients from diverse backgrounds, emphasizing the importance of seeking out these experiences to enhance cultural competence.(2) |
| Cultural knowledge | The active pursuit of understanding different cultural backgrounds, including ethno pharmacology, disease prevalence, and the barriers that specific ethnic groups face in accessing healthcare.(2) |
| Cultural skill | The ability to conduct cultural assessments with sensitivity and appropriateness, using the right tools and techniques.(2) |
| Diversity | The inclusion of individuals from various national origins, ethnicities, religions, socioeconomic backgrounds, sexual orientations, gender identities, and more. In healthcare, it encompasses both the representation of providers from diverse backgrounds in the workforce and the inclusion of patients from diverse backgrounds within the healthcare system.(3,4) |
| Discrimination (in health care) | Inferior healthcare services, unequal treatment or disregard of individuals or groups based on assumptions related to their socio-demographic characteristics, ex. age, gender, sex, race, ethnicity, socioeconomic status, disability, geographic region.(4) |
| Empathy | The ability to understand and share another person's emotions, thoughts, and experiences from their perspective rather than your own. While it doesn't automatically include the urge to help, empathy can lead to feelings like sympathy or personal distress, which may inspire action.(1) |
| Prejudice | Preconceived negative attitude toward an individual or group, formed without prior experience with them and often distorting how we interpret information about them. It involves both *emotional* reactions (e.g., fear, anger, contempt, or hatred) and *cognitive* beliefs, such as stereotypes.(1) |
| Social distance (in healthcare) | Provider's reluctance to engage fully with certain patients, whether through minimizing personal interactions, avoiding touch, or displaying a lack of warmth and empathy.(1) |
| Stigma | The negative social attitude attached to a characteristic of an individual that may be regarded as a mental, physical, or social deficiency.(1) |
| Stereotype | Cognitive generalizations, such as beliefs and expectations, about the attributes and traits of individuals within a group or social category. Stereotypes streamline and speed up perception and judgment.(1) |
| Healthcare providers | Healthcare professionals, including doctors, nurses, pharmacists, therapists, lab scientists, researchers, and public health workers, provide care, diagnose conditions, administer treatments, and offer health management guidance. |
| Marginalized (vulnerable/disadvantaged) groups | Populations that experience social, economic, or political exclusion and inequality due to factors such as race, ethnicity, socioeconomic status, gender, sexual orientation, disability, or other characteristics, resulting to limited access to resources and opportunities compared to more privileged groups.(5) |
| IAT (Implicit Association Test) | Tool used to measure unconscious biases by assessing how quickly individuals associate different concepts, such as social groups and attributes (ex. Black/unpleasant or White/pleasant, overweight/lazy or underweight/healthy), revealing implicit attitudes that may not be consciously recognized. Faster responses when pairing certain concepts together suggest stronger implicit associations.(6) |

**Supplementary file 2. Search String Strategy for Literature Research**

The search strategy for each database is the following:

**PubMed**

("Attitude of Health Personnel"[MeSH Terms] OR "Health Personnel"[MeSH Terms] OR "Health Personnel"[Title/Abstract:~4] OR "Health Care Provider"[Title/Abstract:~3] OR "Health Care Providers"[Title/Abstract:~3] OR "Healthcare Provider"[Title/Abstract:~3] OR "Healthcare Worker"[Title/Abstract:~3] OR "Health Care Professional"[Title/Abstract:~4] OR "Healthcare Providers"[Title/Abstract:~3] OR "Healthcare Workers"[Title/Abstract:~3] OR "Health Care Professionals"[Title/Abstract:~3] OR "Health Professionals"[All Fields] OR "Health Professional"[All Fields] OR "Health Professions"[All Fields] OR "Health care Professions"[All Fields] OR "students, medical"[MeSH Terms] OR "medical students"[Title/Abstract:~3] OR “cancer clinical trials” OR "Clinical Trials as Topic"[MeSH Terms]) AND ("Implicit measures"[All Fields] OR "bias, implicit"[MeSH Terms] OR “Bias, Implicit*” OR "Implicit bias"[Title/Abstract:~4] OR "Subconscious Bias"[Title/Abstract:~4] OR "Hidden Bias"[Title/Abstract:~4] OR "Implicit biases"[Title/Abstract:~4] OR "Subconscious Biases"[Title/Abstract:~4] OR "Hidden Biases"[Title/Abstract:~4] OR "unintentional bias"[Title/Abstract:~4] OR "unintentional biases"[Title/Abstract:~4] OR "unconscious bias"[Title/Abstract:~4] OR "unconscious biases"[Title/Abstract:~4] OR "stereotype*"[MeSH Terms] OR "stereotyping*"[All Fields] OR "implicit stereotype*"[All Fields] OR "Prejudice"[MeSH Terms] OR ("Prejudice"[MeSH Terms] OR "Prejudice"[All Fields] OR "prejudices"[All Fields]) OR (("bias"[MeSH Terms] OR "bias"[All Fields] OR "cultural competency/education"[All Fields] OR "cultural competency/education"[MeSH Terms]) AND (("unconscious, psychology"[MeSH Terms] AND "Healthcare Disparities"[All Fields]) OR "Cultural Diversity"[All Fields] OR "education, medical"[MeSH Terms] OR "Healthcare Disparities"[MeSH Terms] OR "Cultural Diversity"[MeSH Terms] OR "education, medical"[MeSH Terms] OR "Psychology"[MeSH Terms]))) AND ("minimize*"[All Fields] OR "eliminate*"[All Fields] OR "reduce*"[All Fields] OR “modify”[All Fields] OR “piloting” OR "mitigate*"[All Fields] OR “Teaching*” OR “procedure*” OR "Addressing Bias"[Title/Abstract:~4] OR "bias training"[Title/Abstract:~4] OR "Bias Intervention"[Title/Abstract:~4] OR "bias test"[Title/Abstract:~2] OR "Feasibility Studies"[MeSH Terms] OR "Digital Health"[MeSH Terms] OR "education, medical"[MeSH Terms] OR "Health Education"[MeSH Terms] OR "medical education"[Title/Abstract:~3] OR "Health Education"[Title/Abstract:~4] OR "health personnel/education"[MeSH Terms] OR ("educability"[All Fields] OR "educable"[All Fields] OR "educates"[All Fields] OR "education"[MeSH Subheading] OR "education"[All Fields] OR "educational status"[MeSH Terms] OR ("educational"[All Fields] AND "status"[All Fields]) OR "educational status"[All Fields] OR "education"[MeSH Terms] OR "education s"[All Fields] OR "educational"[All Fields] OR "educative"[All Fields] OR "educator"[All Fields] OR "educator s"[All Fields] OR "educators"[All Fields] OR "teaching"[MeSH Terms] OR "teaching"[All Fields] OR "educate"[All Fields] OR "educated"[All Fields] OR "educating"[All Fields] OR "educations"[All Fields])) AND 2004/01/01:2024/12/31[Date - Publication]

**Scopus**

( TITLE-ABS-KEY ( implicit.attitude* OR subconscious.bias* OR hidden.bias* OR implicit.bias* OR ( hidden AND bias* ) OR ( unintentional AND bias* ) OR ( unconscious AND bias* ) OR stereotyp* OR prejudice* OR cultural.competency OR unconscious.psychology OR healthcare.disparities OR cultural.diversity ) AND TITLE-ABS-KEY ( minimiz* OR eliminat* OR reduc* OR mitigat* OR addressing.bias OR address* OR bias.training OR bias.intervention* OR intervention* OR bias.test OR education* OR training OR medical.education OR health.education OR feasibility.studies OR digital.health ) AND TITLE-ABS-KEY ( health.personnel OR healthcare.personnel OR healthcare.provider* OR healthcare.worker* OR healthcare.professional* OR health.professional* OR healthcare.profession* OR medical.students OR cancer.clinical.trials OR clinical.trials ) ) AND PUBYEAR > 2003 AND PUBYEAR < 2025

**Cochrane**

implicit.attitude* OR subconscious.bias* OR hidden.bias* OR implicit.bias* OR subconscious.bias* OR hidden.bias* OR unintentional.bias* OR unconscious.bias* OR stereotyp* OR prejudice* OR cultural.competency OR unconscious.psychology OR healthcare.disparities OR cultural.diversity in Title Abstract Keyword AND minimiz* OR eliminat* OR reduc* OR mitigat* OR addressing.bias OR address* OR bias.training OR bias.intervention* OR intervention* OR bias.test OR education* OR training OR medical.education OR health.education OR feasibility.studies OR digital.health in All Text AND health.personnel OR healthcare.personnel OR healthcare.provider* OR healthcare.worker* OR healthcare.professional* OR health.professional* OR healthcare.profession* OR medical.students OR cancer.clinical.trials OR clinical.trials in Title Abstract Keyword - (Word variations have been searched)

**Table 1. Summary findings of all studies included in the systematic review**

| Author | Year | Study focus | population size (n)* | Population specialty | Intervention type | Assessment tools | Short-term efficacy | Long-term efficacy | Outcome |
| --- | --- | --- | --- | --- | --- | --- | --- | --- | --- |
| Husain et al. | 2023 | healthcare disparities | 14 | physicians | lectures, videos, podcasts, workshops, discussions, additional online resources | questionnaire; open-ended questions | improved | NA | explicit bias, feasibility |
| Koran-Scholl et al. | 2023 | weight bias | 56 | physicians | video as part of a dedicated one-hour didactic on obesity bias | questionnaire; open-ended questions | improved | NA | explicit bias, awareness about one's bias, clinical skills |
| Nabil et al. | 2023 | sexuality/  gender bias | 37 | physicians, nurses | PowerPoint presentations, hands-on training | Homophobia Scale | improved | sustained for doctors but not nurses | explicit and implicit bias, knowledge about bias , satisfaction |
| Pratt-Chapman et al. | 2023 | sexuality/  gender bias | 22 | healthcare providers | educational program, reflection, taking action workshop | Queering Individual and Relational Skills and Knowledge Scales (QUIRKS)-Provider | all factors improved except Attitudes about Sexual and Gender Minorities care | NA | explicit bias, clinical skills |
| Brochu et al. | 2019 | weight bias | 45 | clinical psychology trainees | educational seminar | Anti-fat Attitudes Questionnaire; Attitudes toward fat clients scale | improved | NA | explicit bias |
| Sherf-Dagan et al. | 2022 | weight bias | 249 | healthcare providers | online education module | Anti-Fat Attitudes Questionnaire; short form of fatphobia scale (F-scale); beliefs about the causes of obesity questionnaire | improved | not sustained | explicit bias |
| Tajeu et al. | 2022 | race/  ethnicity bias | 24 | non-physician, non-nursing staff members | online educational modules | IAT; Symbolic Racism Scale | improved | NA | explicit and implicit bias, clinical and communicational skills, feasibility |
| Sabin et al. | 2022 | healthcare disparities | 111 | academic family, internal, and emergency medicine providers | online educational modules | IAT; bias awareness measure (Girod et al.) | improved | NA | explicit and implicit bias |
| Chae et al. | 2021 | cultural competency | 17 | public health workers | online lectures, case studies, brief reflective journaling | Cultural Competence Scale for Nurses-Short Form; Organizational Cultural Competence Measure for Human Service Agencies; Migrant trust and satisfaction measure | improved | sustained | cultural competency, patients trust and satisfaction, feasibility |
| Rodriguez et al. | 2021 | healthcare disparities | 116 | physicians | clinical vignettes, lectures, reflection exercice | modified Classification of Kirkpatrick’s’ Educational Outcomes | improved | NA | explicit bias, knowledge about bias, communicational skills |
| Wijayatunga et al. | 2021 | weight bias | 147 | dietitians | online theory-based video intervention | IAT; Anti-Fat Attitude Test | not improved | NA | explicit and implicit bias, feasibility |
| Seay et al. | 2020 | sexuality/  gender bias | 33 | physicians | educational program, clinical vignettes, reflection exercise | Modern Homonegativity Scale; Physicians’ Attitudes Toward Lesbian, Gay, Bisexual, and Transgender Patients Scale; Gay Affirmative Practice Scale | improved | NA | explicit bias, clinical skills, feasibility |
| Xiao et al. | 2020 | ageism | 113 | nurses | educational program, practical activities, reflection exercise | Clinical Cultural Competency Questionnaire | improved | NA | cultural competency, feasibility |
| Wells et al. | 2017 | cultural competency | 41 | physician investigators and clinical research associates | in- person or online educational program | CCA Tool; Minority Accrual Rates | partially improved cultural competency, no difference in minority accrual rates | NA | cultural competency, rate of recruitment in trials |
| White Hughto et al. | 2021 | sexuality/  gender bias | 34 | healthcare providers | educational program, role-playing activities, discussion | Willingness to provide gender-affirming care survey; Transgender Knowledge, Attitudes, and Beliefs scale; Subjective norms to provide gender-affirming care scale; Transgender Clinical Competence scale | improved | sustained | cultural competency, clinical skills, feasibility |
| Villani et al. | 2017 | mental health bias | 58 | healthcare administrators and students | educational intervention, testimonies of psychiatric services users, movie, discussion | Attitudes to Mental Illness scale | partially improved (9 out of 27 items) | NA | explicit bias |
| Lin et al. | 2020 | cultural competency | 97 | nurses | educational program, narratives, movie, role-playing activities | Nursing Cultural Competence Scale | not improved | not sustained | cultural competency |
| Costa et al. | 2016 | sexuality/  gender bias | 307 | healthcare providers | online educational program, perspective talking and activity planning | Prejudice Against Sexual and Gender Diversity Scale | improved | NA | implicit bias, feasibility |
| Lohiniva et al. | 2016 | HIV-  related stigma | 203 | physicians, nurses | educational intervention, discussions, practical activities | scale for stigmatizing attitudes and fear-based stigma | improved | NA | explicit bias, clinical skills |
| Fox et al. | 2016 | sexuality/  gender bias | 85 | healthcare providers | providers' training adaptation following adapting evidence-based practices to meet local needs | gender sensitivity and knowledge survey | improved | NA | cultural competency, feasibility |
| Ziganshin et al. | 2015 | healthcare disparities | 20 | physicians | international medical exchange programs (clinical experience and education) | survey | improved | NA | cultural competency |
| Tarasoff et al. | 2014 | sexuality/  gender bias | 23 | assisted human reproduction service providers | theater workshop followed by audience intervention | knowledge and comfort questionnaires | knowledge scores significantly improved while comfort scores minimally decreased | NA | explicit bias, clinical skills, feasibility |
| Porter et al. | 2014 | sexuality/  gender bias | 76 | elder service providers | educational program | survey | improved | NA | explicit bias |
| Li et al. | 2013 | HIV-  related stigma | 880 | healthcare providers | popular opinion leaders trained to disseminate stigma reduction messages through conversation with any service providers in the hospitals | questionnaires to measure general prejudicial attitude toward PLH; providers’ avoidance intent and perceived institutional support from the hospital | improved | sustained and strengthened | explicit bias |
| Halm et al. | 2012 | race/  ethnicity bias | 24 | healthcare providers | educational program, critical reflections | Intercultural Development Inventory; Frommelt Attitudes Toward Caring of the Dying; knowledge of cultural beliefs/traditions; self-perceived comfort in providing culturally sensitive end-of-life care | improved understanding of end-of-life care beliefs, preferences, and practices, comfort and effectiveness in providing culturally sensitive end-of-life care | NA | cultural competency, feasibility |
| Stone et al. | 2013 | cultural competency | 73 | healthcare providers | educational intervention, role playing activities, clinical vignettes, action planning | questionnaires | improved | NA | cultural competency |
| Delgado et al. | 2013 | cultural competency | 75 | nurses, healthcare administrator | educational program, simulation, exploring one’s own culture exercise (drawing or writing, game with cards) | IAPCC-R | improved | declined | cultural competency |
| Liekens et al. | 2013 | healthcare disparities | 51 | pharmacists | educational program, role playing activities | Social Distance Scale for Depression; Depression Care Practice Scale; Depression Care Attitude Scale | significant difference in the change in both social distance and practice but no significant difference in the change in attitude between the 2 groups | NA | explicit bias, clinical skills, feasibility |
| Celik et al. | 2012 | healthcare disparities | 31 | healthcare providers | educational program, exercises, Interactive theoretic discussion, action planning | survey for diversity awareness; observation assessments | improved (less effective among nursing home participants) | NA | explicit bias, feasibility |
| McGuire et al. | 2012 | race/  ethnicity bias | 515 | healthcare providers | educational program | questionnaire | improved | NA | cultural competency |
| Palmer et al. | 2011 | cultural competency | 132 | healthcare providers | online educational program, clinical vignettes | questionnaire | improved | NA | cultural competency, feasibility |
| Steed et al. | 2010 | race/  ethnicity bias | 13 | occupational therapists | educational program, activities, reflection exercise | IAT; Racial Argument Scale | not improved | NA | implicit bias |
| Wu et al. | 2008 | HIV-  related stigma | 70 | physicians, nurses, lab technicians | participatory small group activities (role-plays, games, group discussions) and testimony by an HIV advocate | questionnaires | improved | sustained and strengthened | explicit bias, clinical skills |
| Schim et al. | 2006 | cultural competency | 130 | healthcare providers | educational program, discussion | CCA Tool | improved | NA | cultural competency |
| Thom et al. | 2006 | cultural competency | 23 | primary care physicians | educational program, role-playing activities, group exercises, use of trigger tapes, and handouts | Patient-Reported Physician Cultural Competence scale | not improved | NA | cultural competency, patients trust, satisfaction and outcomes |
| Wallace et al. | 2006 | ageism | 18 | nurses | educational program | Kogan's Attitudes Toward Old People Scale; Geriatric Institutional Assessment Profile | improved | NA | explicit bias, clinical skills, feasibility |
| Brathwaite et al. | 2005 | cultural competency | 76 | nurses | educational program, simulation game, cultural bingo game, group discussion, reflection exercise | IAPCC-R | improved | sustained | cultural competency, feasibility |
| Majumdar et al. | 2004 | cultural competency | 40 | nurses, homecare workers | cultural sensitivity training | Dogmatism Scale; Defensiveness Open-Closed Mindedness and Nurses’ Attitude Toward Culturally Different Patients Questionnaire | improved | NA | cultural competency , patients satisfaction and outcomes, feasibility |
| Hayes et al. | 2004 | substance use disorder | 90 | healthcare providers | educational program, group activities and acceptance, mindfulness, and cognitive defusion exercises (ACT training) | Community Attitudes Toward Substance Abusers; Maslach Burnout Inventory. Stigmatizing Attitudes-Believability | improved | sustained only for ACT cohort | explicit bias |
| Ogunyemi et al. | 2021 | healthcare disparities | 103 | physicians | educational program, clinical vignettes and reflective group discussions | surveys and providers' first impressions assessment | improved | NA | implicit bias, knowledge about implicit bias |
| Horky et al. | 2017 | cultural competency | 31 | physicians | online educational modules, clinical vignettes and discussion | questionnaire | improved | NA | cultural competency |
| Barrett et al. | 2022 | race/  ethnicity bias | 119 | healthcare providers | online educational modules combined with facilitated peer-to-peer discussion, action planning | questionnaire | improved | sustained | explicit bias, implicit bias awareness, feasibility |
| Donisi et al. | 2020 | sexuality/  gender bias | 102 | healthcare providers | educational program, reflections, large group discussions, small group activities, role play, videos, case studies | questionnaire | improved | NA | explicit bias, clinical skills |
| Brown-Madan et al. | 2023 | healthcare disparities | 95 | nurses | educational program, patients' testimonies, role play, reflection exercise | survey | improved | sustained | explicit bias, bias awareness, feasibility |
| Nelson et al. | 2015 | race/  ethnicity bias | 19 | physicians | educational program | survey | improved | NA | explicit bias |
| Lewis et al. | 2023 | race/  ethnicity bias | 166 | healthcare providers | Decolonizing methods workshop | Indigenous Knowledge and Beliefs Scale, Scale of Ethno cultural Empathy | improved participants’ Indigenous knowledge and beliefs and aspects of empathy including awareness | NA | explicit bias, empathy, clinical skills |
| Sukhera et al. | 2018 | mental health bias | 69 | physicians, nurses | educational program, role playing, discussions, reflection exercise | Mental Illness Clinician Attitudes scale version 4; Brief Mental Illness Attitudes scale | improved for pediatric emergency providers not in adult emergency providers | not sustained | explicit bis, implicit bias, empathy |
| Mittal et al. | 2020 | mental health bias | 19 | physicians, nurses | contact intervention (clinical vignette) and discussion plus booster session one month after | Opening Minds Scale for healthcare Providers; Attribution Questionnaire; Social Distance Scale | not improved | NA | explicit bias |
| Perales-Puchalt et al. | 2022 | mental health bias | 44 | healthcare providers | educational program, discussion | General Practitioners Confidence and Attitude Scale for Dementia | improved | NA | explicit bias, clinical skills, feasibility |
| Centola et al. | 2021 | healthcare disparities | 80 | physicians | real-time information exchange in structured clinicians' networks | questionnaire | improved | NA | explicit bias, clinical skills |
| Fair et al. | 2021 | cultural competency | 35 | midwives | culturally sensitive maternity care training | Groningen Reflection Ability Scale | improved | NA | cultural competency, clinical skills, feasibility |
| Barnabe et al. | 2021 | race/  ethnicity bias | 16 | physicians | experiential learning workshops with real-time feedback (skill-based teaching intervention, lectures, role playing, case studies, facilitator training) | Social Cultural Confidence in Care Survey; survey about self-reported strategies to address social issues and improve therapeutic relationships and engagement with teaching others | improved | sustained | cultural competency, explicit bias, communication skills, patients experience and outcomes, feasibility |
| Holman et al. | 2020 | sexuality/  gender bias | 43 | healthcare providers | educational program, role playing activities | LGBT supportive attitudes measure (LaMar & Kite, 1998); LGBT knowledge questionnaire; Perceptions of preparedness survey | improved | NA | cultural competency, clinical skills |
| Davtyan et al. | 2019 | HIV-  related stigma | 38 | healthcare providers | educational program, photovoice intervention (contact with PLH personal stories), clinical vignettes, discussion | questionnaire | improved knowledge and attitudes but not stigma | not sustained | explicit bias, implicit bias, clinical skills |
| Filmer et al. | 2019 | cultural competency | 58 | nurses | educational program, role-playing activities, experiential exercises and activities, discussions and reflection exercise | case vignettes, semi-standardized interviews, opportunistic observation assessments | self‐reports showing positive but mostly not significant improvement and objective assessments mostly showing significant positive changes | NA | cultural competency |
| Omori et al. | 2012 | mental health bias | 51 | physicians | clinical training period in psychiatry | IAT; Link's devaluation-discrimination scale | negative impact | NA | implicit bias |
| Puri Singh et al. | 2016 | healthcare disparities | 96 | physicians, nurses | online video-based educational intervention | General Perceptions About Sickle Cell Patients Scale | improved | sustained | explicit bias |
| Flanagan et al. | 2016 | healthcare disparities | 14 | healthcare providers | photovoice intervention (witnessing people's recovery stories) | Characteristics Scale and Affective Reaction Scale; Social Distance Scale; Attribution Questionnaire; Recovery Knowledge Inventory and Competence Assessment Instrument | improved | NA | explicit bias, implicit bias, feasibility |
| Hirsh et al. | 2019 | healthcare disparities | 49 | physicians | online perspective-taking intervention (personalized feedback about one's own bias, real-time dynamic interactions with virtual patients, and videos depicting how pain impacts the patients’ lives) | questionnaire | improved | NA | explicit bias, implicit bias, empathy |
| Hawke et al. | 2014 | mental health bias | 32 | healthcare providers, patients and public | intergroup contact theory and narrative medicine (clinical vignette video) | Mental Illness Stigma Scale; Social Distance Scale; Mental Illness: Clinicians’ Attitudes Scale | improved mainly among providers | sustained among providers | explicit bias, feasibility |
| Reddyhough et al. | 2021 | mental health bias | 59 | healthcare providers | educational program, dissemination of information about voice hearing, clinical vignettes, role-playing activities, discussion | IAT; Social Distance Scale; Attribution Questionnaire-20 | improved explicit stigma but not implicit stigma | NA | explicit bias, implicit bias |
| Browne et al. | 2018 | healthcare disparities | 43 | healthcare providers | educational program and discussion | survey; open-ended interviews; observational assessment | improved | NA | explicit bias, clinical skills, feasibility |
| Wyckoff et al. | 2018 | sexuality/  gender bias | 30 | nurses | educational presentation | Gay Affirmative Practice Scale | improved behavior subscale but not beliefs subscale of GAP scale | NA | cultural competency |
| Akhetuamhen et al. | 2022 | cultural competency | 63 | physicians | educational program and discussions | questionnaire | improved | NA | cultural competency |
| Rhoten et al. | 2022 | sexuality/  gender bias | 420 | healthcare providers | educational program (videos, handouts, activity sheets) | questionnaire | improved | NA | cultural competency |
| Akearok et al. | 2020 | race/  ethnicity bias | 18 | healthcare providers | orientation mobile application (long-form text, short descriptions, lists, quizzes, and aggregation of community data (such as cost of living, availability of resources, important phone numbers)) | feasibility assessment; interviews; observations and electronic feedback | feasibility good | NA | cultural competency, feasibility |
| Khan et al. | 2019 | race/  ethnicity bias | 15 | healthcare providers | online educational low-fidelity serious game | Questionnaire for User Interaction Satisfaction; Game Engagement Questionnaire; open-ended questions | feasibility good | NA | feasibility |
| Nyblade et al. | 2020 | HIV-  related stigma | 599 | healthcare providers | educational training | questionnaire | improved | NA | explicit bias |
| Sukhera et al. | 2019 | mental health bias | 21 | physicians | IAT, picture drawing and interview about their picture and experience of taking their IAT | semi-structured interview; open ended questions | improved | NA | implicit bias awareness |
| Alarcão et al. | 2022 | cultural competency | 50 | healthcare providers | educational program, skill-building activities | feasibility assessment | feasibility good | NA | feasibility |
| Neff et al. | 2020 | healthcare disparities | 275 | physicians | educational program and discussion | open-ended, written-response surveys | feasibility good, feeling reconnected to their original motivations for entering the health professions | NA | explicit bias, feasibility |
| Burgess et al. | 2018 | race/  ethnicity bias | 280 | healthcare providers | narratives, online educational resources | questionnaire | improved | sustained | explicit bias, implicit bias |
| Wasmuth et al. | 2023 | healthcare disparities | 51 | healthcare providers | filmed narrative theater-based training, reflection exercise | Acceptance and Action Questionnaire–Stigma | improved | NA | explicit bias, implicit bias |
| Roberts et al. | 2023 | healthcare disparities | 21 | healthcare providers | online educational program, discussion, behavior change activity activities, reflection exercise | questionnaires | improved | NA | explicit bias, feasibility |
| Hershberger et al. | 2022 | healthcare disparities | 158 | healthcare providers | online simulation training | questionnaire | improved | NA | explicit bias, implicit bias, empathy |
| Wu et al. | 2009 | healthcare disparities | 1259 | public health workers | educational program | Attribution Questionnaire - Short Form - 8 Items (AQ-S8) for Measures of Illness Stigma | improved | NA | explicit bias, clinical skills |
| Bristol et al. | 2018 | sexuality/  gender bias | 40 | healthcare providers | online educational program (presentations, interactive exercises, small-group discussions, and short films) | Ally Identity Measure | improved | NA | explicit bias, clinical skills |
| Long et al. | 2022 | sexuality/  gender bias | 56 | healthcare providers | storytelling events, reflection exercise | modified Jefferson Scale of Physician Empathy; modified Jefferson Scale of Patient’s Perceptions of Physician Empathy | improved | NA | explicit bias, empathy, patients perception |
| Aggarwal et al. | 2018 | cultural competency | 423 | healthcare providers | online Cultural Formulation Interview training (active learning with CFI along with passive didactic lectures-videos) | survey and closed-ended questionnaire | feasibility good | NA | explicit bias, clinical skills, feasibility |
| Wheeler et al. | 2018 | mental health bias | 566 | pharmacy staff | online experiential learning techniques (narratives, role-playing activities, PowerPoint presentations, interactive case vignettes and panel discussions) | Depression Attitude Questionnaire; Mental Illness Attitude Scale; questionnaire to self-assess confidence, skills, motivations and barriers to working with consumers and carers with mental health-related issues | improved | NA | explicit bias, implicit bias, clinical skills |
| Chapman et al. | 2018 | race/  ethnicity bias | 69 | physicians | Photovoice visual intervention (narrative photography), discussion, reflection exercise | Ethno cultural Empathy Scale, Jefferson Physician Empathy Scale, Patient-Practitioner Orientation Scale, Affect Misattribution Procedure | mixed | NA | implicit bias, empathy |
| Sempértegui et al. | 2018 | race/  ethnicity bias | 20 | mental healthcare professionals | educational program and practical interventions (practical and imaginary exercises, role-playing activities, case evaluations, plenary discussions, and homework) | Attitude-Awareness, Skills and Knowledge scale; Diversity Competence Knowledge Test | improved | sustained | implicit bias awareness, clinical skills |
| Rosendale et al. | 2017 | healthcare disparities | 20 | physicians | educational program, IAT | survey | improved | NA | implicit bias, cultural competency |
| Durey et al. | 2017 | race/  ethnicity bias | 39 | healthcare providers | educational program, case studies and group discussions | questionnaire; open-ended questions | improved | sustained | cultural competency, feasibility |
| Cruz-Oliver et al. | 2017 | race/  ethnicity bias | 74 | healthcare providers | video soap opera (telenovela) along with the standard educational seminar | questionnaire | improved | NA | cultural competency, feasibility |
| Pepping et al. | 2018 | sexuality/  gender bias | 96 | mental health professionals | educational program, group discussion, reflection exercises | Lesbian, Gay, and Bisexual Affirmative Counseling Self-Efficacy Inventory; Modern Homo-negativity Scale; Acceptance and Action Questionnaire-Stigma | improved | NA | explicit bias, implicit bias awareness, clinical skills |
| Lam et al. | 2015 | mental health bias | 66 | physicians | educational program (postgraduate training in Community Psychological Medicine) | questionnaire, observational assessment | improved mainly for attitudes towards depression rather than schizophrenia | NA | explicit bias, implicit bias |
| Perry et al. | 2015 | cultural competency | 60 | nurses and allied health professionals | online simulation training | questionnaire; open-ended questions | improved, feasibility good | NA | explicit bias, clinical skills, feasibility |
| Owiti et al. | 2014 | cultural competency | 28 | mental health professionals | narrative-based cultural consultation service (ethnographic interview method to assess patients in the presence of referring clinicians) | Tool for Assessing Cultural Competence Training; open-ended questions | improved | NA | cultural competency, feasibility |
| Grady et al. | 2014 | race/  ethnicity bias | 15 | nurses | educational program | IAPCC-R | improved | NA | cultural competency, feasibility |
| Park et al. | 2013 | cultural competency | NA | nurses | online educational program (lecture & interactive case scenario, quiz video & audio, discussion, individual and team project) | questionnaire | feasibility good, users satisfied | NA | feasibility |
| Kutob et al. | 2013 | cultural competency | 49 | physicians | interactive skills-based training on clinical vignettes | CCA Tool | improved subscales measuring physicians' nonjudgmental attitudes and elicitation of patients' beliefs, but not no significant differences on total CCAT score or subscales measuring cultural knowledge, nonverbal communication, and empowerment | NA | cultural competency |
| Michaels et al. | 2012 | cultural competency | 1285 | healthcare providers | educational program | questionnaire patients recruitment rate assessment | improved | sustained | explicit bias, cultural competency, clinical skills, communication skills |
| Liu et al. | 2022 | race/  ethnicity bias | 11 | school-based mental health clinicians | educational program, IAT, case-based learning | Concern for Discrimination Scale; Intervention Usability Scale; Implicit Bias Knowledge Quiz | improved | sustained | implicit bias and awareness, feasibility |
| Khanna et al. | 2009 | cultural competency | 43 | physicians, nurses, healthcare administrators | educational program | CCA Tool | improved | NA | cultural competency |
| Elminowski et al. | 2015 | cultural competency | 18 | nurses | educational program, self-assessment tool of cultural beliefs, case study | CCA Tool | improved | NA | cultural competency, feasibility |
| Li et al. | 2015 | mental health bias | 40 | healthcare providers | educational program, clinical practice | Mental Health Knowledge Schedule; Mental illness: Clinicians’ Attitudes; Reported and Intended Behaviour Scale | improved | sustained | explicit bias, clinical skills |
| Jones et al. | 2021 | weight bias | 27 | physiotherapists | educational seminar | Attitude Towards Obese Persons; Beliefs About Obese Persons | not improved | NA | explicit bias |
| Eiroa-Orosa et al. | 2021 | mental health bias | Six primary care centers, 185 randomized, 128 in intervention group, 88 follow up session, 3 months follow up survey | primary care providers | educational program, discussions, patients' narratives, providers' self-diagnosis session, self-organized activities, follow-up session 1 month later to reinforce the concepts learned and share the progress of the self-organized activities | Opening Minds Stigma Scale for healthcare providers | not improved | not sustained | explicit bias, implicit bias awareness |
| Eiroa-Orosa et al. | 2021 | mental health bias | six mental health centers, 186 randomized, 113 in the intervention group, 3 months follow up survey | mental health providers | educational program, discussions, patients' narratives, providers' self-diagnosis session, self-organized activities, follow-up session 1 month later to reinforce the concepts learned and share the progress of the self-organized activities | Beliefs and Attitudes towards Mental Health Service users’ rights | improved | not sustained | explicit bias, implicit bias awareness |
| Kennedy-Hendricks et al. | 2022 | substance use disorder | 1842 | healthcare providers | visual campaign with or without an accompanying narrative patient's vignette | questionnaire; feeling thermometer (to measure level of warmth towards OUD people) | improved | NA | explicit bias |
| Geibel et al. | 2016 | HIV-  related stigma | 300 | healthcare providers | educational lecture plus interactive interventions of 1-day supplemental training on stigma at 6 months (pictures based on personal stories of stigma are viewed, using colored cards and markers followed by discussions and reflection activity) | questionnaire for providers' stigma attitude; patients' satisfaction survey | improved | NA | explicit bias , patients satisfaction |
| Zeidan et al. | 2024 | healthcare disparities | 21 | physicians | educational program, IAT and discussion | questionnaire; providers' satisfaction survey | improved, satisfied | NA | implicit bias awareness, feasibility |
| Svetkey et al. | 2019 | race/ ethnicity bias | 37 | healthcare providers | educational presentations, group discussions, video simulations, group exercises, role-playing activity, reflection exercise, changes in clinical practice | questionnaire; open-ended questions; providers' satisfaction survey | improved bias, satisfied | NA | implicit bias, feasibility |
| Glen et al. | 2024 | race/ ethnicity bias | 273 | Academic faculty members including physicians and non | six-hour elective continuing education series; discussion sessions | Questionnaire; open-ended questions | improved | NA | cultural competence |
| Svatkey et al. | 2024 | race/ ethnicity bias | 37 | physician and non-physician clinicians | Didactic presentations, group discussion, video testimonials, group experiential exercise, role-play | Survey; open ended-questions; program evaluation | improved, feasible | NA | implicit bias, feasibility |
| Skoko Rodriguez et al. | 2024 | Weight Bias | 30 | healthcare providers | didactic presentations, short readings, patient stories, group discussions | IAT; Attitudes Toward Obese Persons Scale; Beliefs About Obese Persons Scales ; | improved | sustained | implicit bias |
| Yang et al. | 2024 | HIV-related bias | 406 | nurses | educational sessions, group discussion | Questionnaire; general prejudicial attitude scale ; avoidance behavior scale | improved | sustained | explicit and implicit bias, |
| Mulchan et al. | 2024 | race/ ethnicity bias | 36 | healthcare providers | Live virtual didactic sessions and discussion | Race IAT; explicit SCD bias; programme evaluation | no statistically significant differences between arms, feasible |  | explicit and implicit bias, feasibility |
| Gibbons et al. | 2024 | race/ ethnicity bias | 25 | healthcare providers | racial bias training, educational material and discussions | questionnaire | Improved, feasible | sustained | implicit bias, changes in provider knowledge, feasibility |

*Number (n) of patients completed pre and post test

ACT, Acceptance and Commitment Training; CCA Tool, Cultural Competence Assessment Tool; CFI, Cultural Formulation Interview; HIV, human immunodeficiency virus; IAT, Implicit Association Test; IAPCC (-R), Inventory for Assessing the Process of Cultural Competence Among Healthcare Professionals (-Revised); LGBT, lesbian, gay, bisexual, and transgender; NA not assessed; OUD, opioid use disorder; PLH, people living with HIV

Note 1: satisfaction counted in feasibility assessment

Note 2: knowledge about bias considered as part of explicit or implicit bias evaluated in each study

Note 3: skills referring to either clinical skills or communication skills, or empathy

Note 4: awareness about implicit bias assessed as part of implicit bias changes

**Supplementary Table 2. Interventions focusing on Clinical Trials**


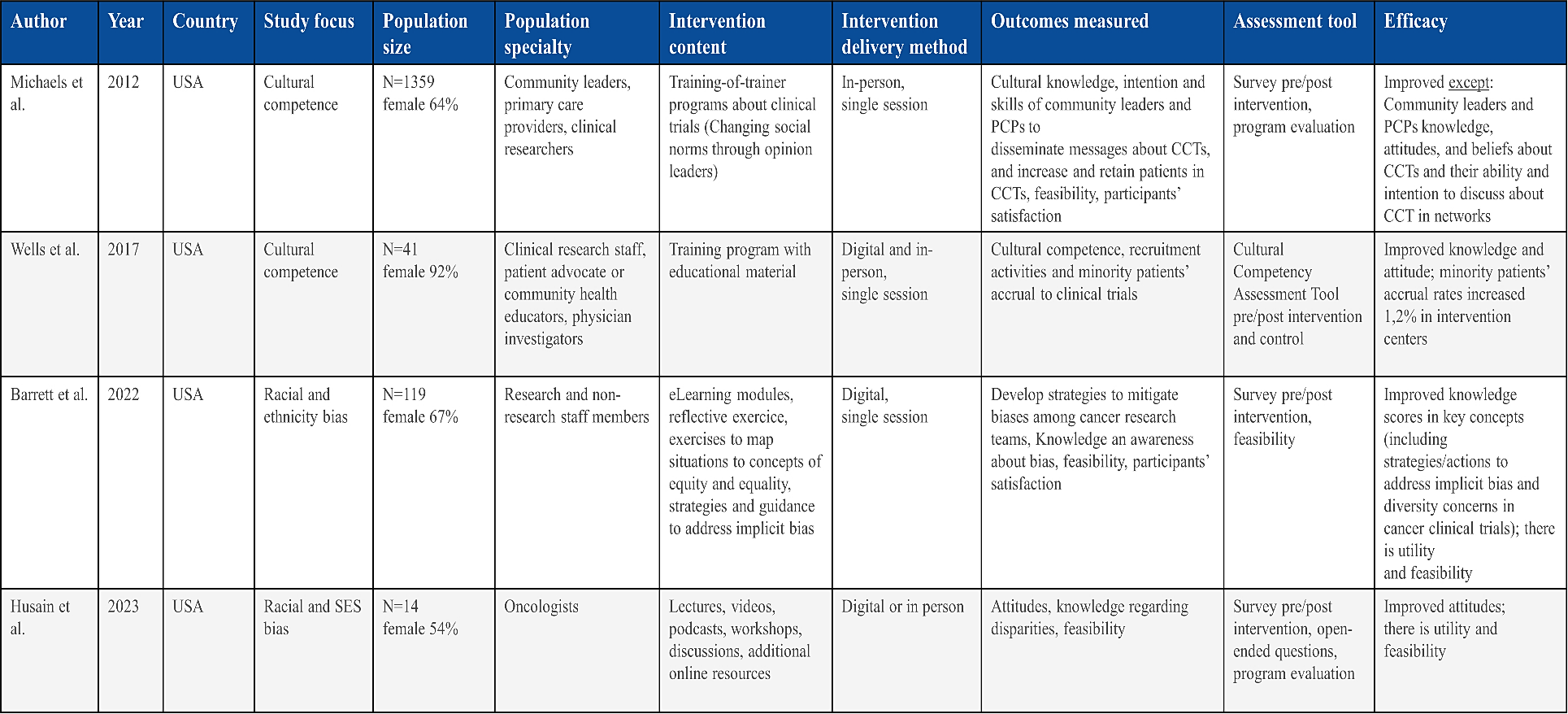


**Supplementary Table 3. Risk of bias of non-randomized trials using (ROBINS I tool)**

|  | Bias due to Confounding | Bias in Selection of Participants | Bias in Classification of Intervention | Bias due to Deviations from Intended Interventions | Bias due to Missing Outcome Data | Bias in Measurement of Outcomes | Bias in Selection of the Reported Result |  |
| --- | --- | --- | --- | --- | --- | --- | --- | --- |
| Author | D1 | D2 | D3 | D4 | D5 | D6 | D7 | Overall |
| Husain et al. | Serious | Moderate | Low | Low | Low | Moderate | Low | Serious |
| Koran-Scholl et al. | Moderate | Low | Low | Low | Moderate | Moderate | Low | Moderate |
| Pratt-Chapman et al. | Serious | Serious | Low | Moderate | Moderate | Moderate | Low | Serious |
| Brochu et al. | Serious | Moderate | Moderate | Moderate | Moderate | Serious | Moderate | Serious |
| Tajeu et al. | Moderate | Moderate | Low | Low | Moderate | Moderate | Low | Moderate |
| Sabin et al. | Serious | Serious | Low | Low | Moderate | Moderate | Low | Serious |
| Rodriguez et al. | Low | Moderate | Low | Low | Moderate | Low | Low | Moderate |
| Seay et al. | Low | Moderate | Low | Low | Moderate | Low | Low | Moderate |
| Xiao et al. | Low | Moderate | Low | Low | Low | Low | Low | Moderate |
| Wells et al. | Moderate | Moderate | Low | Serious | Serious | Low | Moderate | Serious |
| White Hughto et al. | Moderate | Moderate | Low | Moderate | Moderate | Serious | Low | Moderate |
| Villani et al. | Moderate | Moderate | Low | Low | Moderate | Low | Moderate | Moderate |
| Costa et al. | Moderate | Moderate | Low | Low | Low | Serious | Low | Moderate |
| Lohiniva et al. | Moderate | Moderate | Low | Low | Moderate | Moderate | Low | Moderate |
| Ziganshin et al. | Moderate | Moderate | Low | Low | Low | Moderate | Low | Moderate |
| Tarasoff et al. | Moderate | Moderate | Low | Moderate | Moderate | Moderate | Low | Moderate |
| Porter et al. | Serious | Serious | Low | Low | Low | Moderate | Low | Serious |
| Halm et al. | Moderate | Moderate | Low | Low | Low | Moderate | Low | Moderate |
| Stone et al. | Moderate | Moderate | Low | Low | Low | Moderate | Low | Moderate |
| Delgado et al. | Moderate | Moderate | Low | Low | Moderate | Moderate | Low | Moderate |
| Celik et al. | Moderate | Moderate | Low | Low | Moderate | Moderate | Low | Moderate |
| McGuire et al. | Moderate | Serious | Moderate | Moderate | Serious | Moderate | Low | Serious |
| Palmer et al. | Low | Moderate | Low | Low | Serious | Moderate | Low | Serious |
| Steed et al. | Moderate | Serious | Low | Moderate | Moderate | Serious | Moderate | Serious |
| Schim et al. | Serious | Moderate | Low | Moderate | Moderate | Moderate | Low | Serious |
| Wallace et al. | Moderate | Serious | Low | Low | Low | Moderate | Moderate | Serious |
| Brathwaite et al. | Moderate | Serious | Low | Low | Low | Moderate | Low | Serious |
| Ogunyemi et al. | Moderate | Serious | Low | Low | Moderate | Moderate | Low | Serious |
| Horky et al. | Low | Moderate | Low | Low | Moderate | Moderate | Low | Moderate |
| Barrett et al. | Low | Moderate | Low | Low | Low | Moderate | Low | Moderate |
| Donisi et al. | Moderate | Serious | Low | Low | Moderate | Moderate | Low | Serious |
| Brown-Madan et al. | Moderate | Serious | Low | Low | Moderate | Moderate | Low | Serious |
| Nelsonet al. | Moderate | Serious | Low | Low | Moderate | Moderate | Low | Serious |
| Lewis et al. | Moderate | Serious | Low | Low | Moderate | Moderate | Low | Serious |
| Sukhera et al. (2019) | Moderate | Serious | Low | Moderate | Moderate | Moderate | Low | Serious |
| Perales-Puchalt et al. | Serious | Moderate | Low | Moderate | Moderate | Moderate | Low | Serious |
| Fair et al. | Serious | Moderate | Low | Moderate | Moderate | Moderate | Low | Serious |
| Barnabe et al. | Moderate | Moderate | Low | Low | Moderate | Moderate | Low | Moderate |
| Holman et al. | Moderate | Low | Low | Low | Moderate | Moderate | Low | Moderate |
| Davtyan et al. | Moderate | Moderate | Low | Low | Moderate | Moderate | Low | Moderate |
| Filmer et al. | Moderate | Serious | Low | Low | Moderate | Moderate | Low | Serious |
| Omori et al. | Serious | Moderate | Low | Moderate | Low | Moderate | Moderate | Serious |
| Puri Singh et al. | Moderate | Moderate | Low | Low | Low | Moderate | Low | Moderate |
| Hawke et al. | Moderate | Moderate | Low | Low | Moderate | Moderate | Low | Moderate |
| Reddyhough et al. | Moderate | Moderate | Low | Low | Low | Moderate | Low | Moderate |
| Browne et al. | Moderate | Moderate | Low | Low | Moderate | Low | Low | Moderate |
| Wyckoff et al. | Moderate | Moderate | Low | Low | Moderate | Low | Low | Moderate |
| Akhetuamhen et al. | Moderate | Moderate | Low | Low | Moderate | Moderate | Low | Moderate |
| Rhoten et al. | Moderate | Moderate | Low | Moderate | Low | Moderate | Low | Moderate |
| Akearok et al. | Moderate | Moderate | Low | Moderate | Moderate | Moderate | Low | Moderate |
| Khan et al. | Low | Moderate | Low | Low | Low | Moderate | Low | Moderate |
| Nyblade et al. | Moderate | Moderate | Low | Low | Low | Moderate | Low | Moderate |
| Sukhera et al. (2018) | Low | Moderate | Low | Low | Low | Moderate | Low | Moderate |
| Alarcão et al. | Moderate | Moderate | Low | Low | Moderate | Moderate | Low | Moderate |
| Neff et al. | Moderate | Moderate | Low | Low | Low | Moderate | Low | Moderate |
| Wasmuth et al. | Moderate | Moderate | Low | Low | Low | Moderate | Low | Moderate |
| Roberts et al. | Moderate | Moderate | Low | Low | Low | Moderate | Low | Moderate |
| Hershberger et al. | Moderate | Moderate | Low | Low | Low | Moderate | Low | Moderate |
| Wu P.S. et al. | Moderate | Moderate | Low | Low | Low | Moderate | Low | Moderate |
| Bristol et al. | Moderate | Moderate | Low | Low | Moderate | Moderate | Low | Moderate |
| Long et al. | Moderate | Moderate | Low | Low | Moderate | Moderate | Low | Moderate |
| Aggarwal et al. | Serious | Moderate | Low | Low | Moderate | Moderate | Low | Moderate |
| Wheeler et al. | Moderate | Low | Low | Low | Moderate | Low | Low | Moderate |
| Chapman et al. | Moderate | Moderate | Low | Low | Moderate | Low | Low | Moderate |
| Sempértegui et al. | Moderate | Low | Low | Low | Moderate | Low | Low | Moderate |
| Rosendale et al. | Moderate | Low | Low | Low | Moderate | Moderate | Low | Moderate |
| Durey et al. | Moderate | Moderate | Low | Low | Serious | Moderate | Low | Moderate |
| Pepping et al. | Moderate | Low | Low | Low | Low | Moderate | Low | Moderate |
| Lam et al. | Moderate | Low | Low | Low | Moderate | Moderate | Low | Moderate |
| Perry et al. | Moderate | Low | Low | Moderate | Moderate | Serious | Moderate | Serious |
| Owiti et al. | Moderate | Moderate | Low | Moderate | Serious | Serious | Moderate | Serious |
| Grady et al. | Moderate | Moderate | Low | Low | Moderate | Serious | Moderate | Moderate |
| Park et al. | Low | Low | Low | Low | Low | Moderate | Low | Moderate |
| Kutob et al. | Low | Low | Low | Low | Low | Moderate | Low | Moderate |
| Michaels et al. | Moderate | Moderate | Low | Low | Serious | Moderate | Low | Moderate |
| Liu F.F. et al. | Moderate | Moderate | Low | Low | Serious | Moderate | Low | Moderate |
| Khanna et al. | Moderate | Moderate | Low | Low | Serious | Moderate | Low | Moderate |
| Elminowski et al. | Moderate | Moderate | Low | Low | Moderate | Moderate | Low | Moderate |
| Li J. et al. | Moderate | Low | Low | Low | Moderate | Low | Low | Moderate |
| Jones et al. | Serious | Serious | Low | Moderate | Low | Moderate | Moderate | Serious |
| Geibel et al. | Serious | Moderate | Low | Moderate | Low | Moderate | Moderate | Serious |
| Zeidan et al. | Moderate | Low | Low | Moderate | Low | Moderate | Moderate | Moderate |
| Svetkey et al. | Moderate | Moderate | Low | Moderate | Moderate | Moderate | Moderate | Moderate |
| Glenn et al. | Serious | Serious | Low | Moderate | Serious | Moderate | Moderate | Serious |
| Skoko Rodriguez et al. | Serious | Serious | Low | Moderate | Serious | Moderate | Moderate | Serious |
| Yang et al. | Moderate | Low | Low | Serious | Low | Serious | Moderate | Serious |
| Gibbons et al. | Serious | Moderate | Low | Low | Moderate | Moderate | Moderate | Serious |

**Supplementary Table 4. Risk of bias of randomized trials using (RoB 2 tool)**

|  | Bias due to randomization | Bias due to deviations from intended interventions | Bias due to missing outcome data | Bias in measurement of the outcome | Bias in selection of the reported result |  |
| --- | --- | --- | --- | --- | --- | --- |
| Author | D1 | D2 | D3 | D4 | D5 | Overall |
| Nabil et al. | low | moderate | moderate | moderate | low | moderate |
| Sherf-Dagan et al. | low | low | serious | low | low | moderate |
| Chae et al. | low | low | moderate | low | low | moderate |
| Wijayatunga et al. | low | low | serious | moderate | low | serious |
| Lin et al. | moderate | low | low | moderate | low | moderate |
| Fox et al. | moderate | low | serious | moderate | low | serious |
| Li et al. | low | low | low | moderate | low | moderate |
| Liekens et al. | moderate | low | moderate | moderate | low | moderate |
| Wu S. et al. | moderate | moderate | low | moderate | low | moderate |
| Thom et al. | serious | low | serious | moderate | low | serious |
| Majumdar et al. | moderate | serious | serious | moderate | low | serious |
| Hayes et al. | moderate | serious | moderate | moderate | low | serious |
| Mittal et al. | moderate | moderate | moderate | low | low | moderate |
| Centola et al. | low | moderate | moderate | low | low | moderate |
| Flanagan et al. | moderate | moderate | moderate | low | low | moderate |
| Hirsh et al. | moderate | moderate | serious | low | low | serious |
| Burgess et al. | low | low | moderate | low | low | moderate |
| Cruz-Oliver et al. | serious | low | moderate | moderate | low | serious |
| Eiroa-Orosa et al. | low | low | moderate | moderate | low | moderate |
| Kennedy-Hendricks et al. | low | low | moderate | low | low | moderate |
| Mulchan et al. | low | moderate | moderate | moderate | moderate | moderate |

**Supplementary Figure 1. Study outcomes assessed across the studies.**
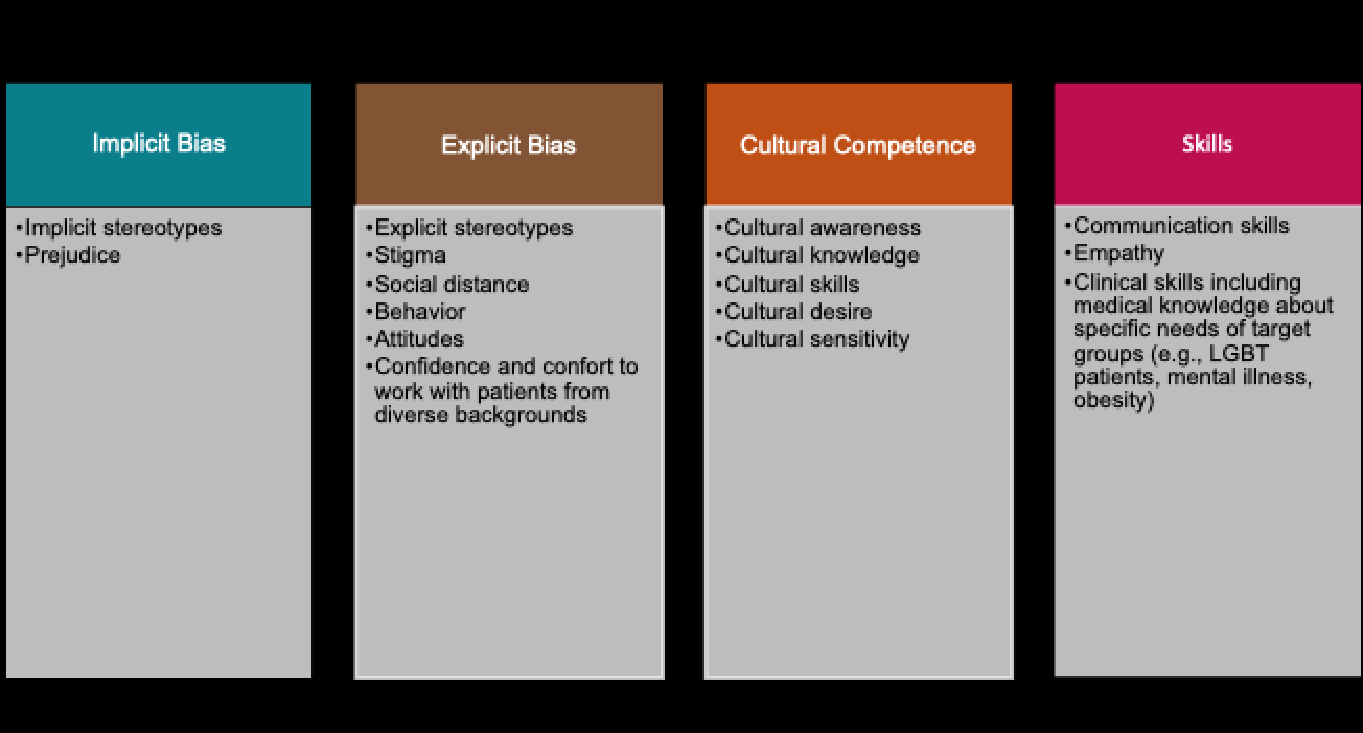


**Supplementary Figure 2. Trends in Outcomes Assessed among studies evaluating Interventions to Reduce Healthcare Providers’ Bias over the years.**

**
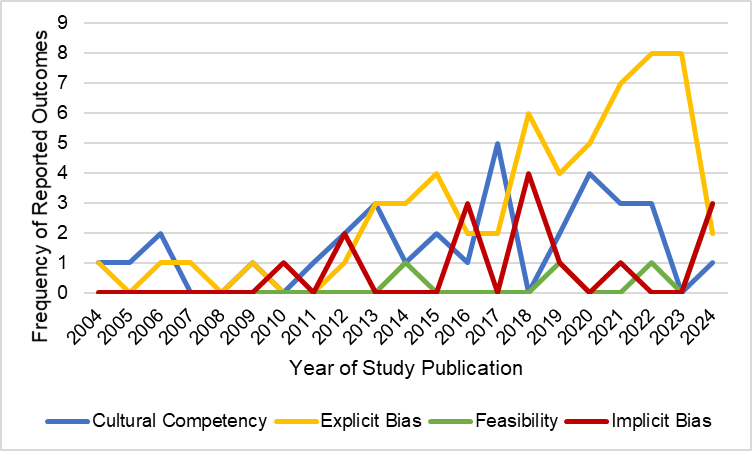
**
